# Multiple origins of endosymbionts in Chlorellaceae with no reductive effects on the plastid or mitochondrial genomes

**DOI:** 10.1038/s41598-017-10388-w

**Published:** 2017-08-30

**Authors:** Weishu Fan, Wenhu Guo, James L. Van Etten, Jeffrey P. Mower

**Affiliations:** 1 Center for Plant Science Innovation, University of Nebraska, Lincoln, NE 68588 USA; 2 Department of Agronomy and Horticulture, University of Nebraska, Lincoln, NE 68583 USA; 3 Wuhan Frasergen Bioinformatics Co. Ltd., Wuhan, Hubei 430075 China; 4 Department of Plant Pathology and Nebraska Center for Virology, University of Nebraska, Lincoln, NE 68583 USA

## Abstract

Ancient endosymbiotic relationships have led to extreme genomic reduction in many bacterial and eukaryotic algal endosymbionts. Endosymbionts in more recent and/or facultative relationships can also experience genomic reduction to a lesser extent, but little is known about the effects of the endosymbiotic transition on the organellar genomes of eukaryotes. To understand how the endosymbiotic lifestyle has affected the organellar genomes of photosynthetic green algae, we generated the complete plastid genome (plastome) and mitochondrial genome (mitogenome) sequences from three green algal endosymbionts (*Chlorella heliozoae*, *Chlorella variabilis* and *Micractinium conductrix*). The mitogenomes and plastomes of the three newly sequenced endosymbionts have a standard set of genes compared with free-living trebouxiophytes, providing no evidence for functional genomic reduction. Instead, their organellar genomes are generally larger and more intron rich. Intron content is highly variable among the members of *Chlorella*, suggesting very high rates of gain and/or loss of introns during evolution. Phylogenetic analysis of plastid and mitochondrial genes demonstrated that the three endosymbionts do not form a monophyletic group, indicating that the endosymbiotic lifestyle has evolved multiple times in Chlorellaceae. In addition, *M. conductrix* is deeply nested within the *Chlorella* clade, suggesting that taxonomic revision is needed for one or both genera.

## Introduction

It is well established that the transition to an obligate endosymbiotic lifestyle often results in massive genomic reduction, particularly in ancient and obligate endosymbiont-host relationships. Most notably, endosymbiotic theory posits that mitochondria and plastids are descendants of α-proteobacterial and cyanobacterial endosymbionts whose genomes likely contained thousands of genes, whereas the mitochondrial genomes (mitogenomes) and plastid genomes (plastomes) of living eukaryotes are small and typically retain a few dozen to a few hundred genes^1, 2^. Numerous additional studies have reported massive genomic reduction from other ancient bacterial endosymbionts, some of which possess genomes under 200 kb in size^3–5^, much smaller compared with their free-living relatives.

Among eukaryotes, several green and red algal lineages are in ancient endosymbiotic relationships with other eukaryotes due to their photosynthetic abilities, leading to a more complicated pattern of gene retention and loss within the endosymbiont genomes. In the most extreme examples, the algal endosymbiont has been reduced to a secondary plastid, resulting in the retention of the algal plastome while the remnant nuclear genome lost most of its genes and the algal mitochondrion was lost completely^6, 7^. Reduction of the plastome can occur if the endosymbionts are no longer required to be photosynthetic, as exemplified by the highly reduced genome of the nonphotosynthetic apicoplast in the malarial parasite *Plasmodium falciparum* ^8^.

Emerging evidence has shown that genomic reduction is also a frequent outcome for endosymbionts in more recent and/or facultative endosymbiotic relationships. For example, the chromatophore (an obligate endosymbiotic cyanobacterium) of the amoeba *Paulinella chromatophora* was established as a novel photosynthetic organelle ~100 million years ago, and this chromatophore genome has experienced extensive genomic reduction, retaining only 26% of the genes compared with its free-living relative^9, 10^. Genomic reduction is also apparent in several different lineages of bacteria that live as facultative endosymbionts of insects^11–14^. The genomes of these endosymbiotic bacteria are usually smaller than their free-living relatives but larger than those of related obligate endosymbionts. These patterns suggest a progressive degradation of endosymbiont genomes over time coupled with an increased reliance on hosts for protection and resources, which likely facilitates a transition from a facultative to an obligate endosymbiotic relationship.

For eukaryotic endosymbionts, less is known about this evolutionary transition from facultative to obligate endosymbiosis. The Trebouxiophyceae, a class of green algae in the Chlorophyta, is an apt system to address this issue because its members have a range of lifestyles, comprising symbionts of fungi to form lichens (e.g. *Trebouxia* and *Myrmecia*), photosynthetic symbionts in ciliates, heliozoans and invertebrates (e.g. *Chlorella* and *Micractinium*), nonphotosynthetic species (e.g. *Prototheca* and *Helicosporidium*) and many free-living representatives^15–20^. One well-studied endosymbiotic association involves the ciliate *Paramecium bursaria* and several species of green algae in *Chlorella*. The relationship is mutualistic in that the *Chlorella* endosymbionts export substantial amounts of photosynthates, particularly maltose, for use by the *Paramecium* host^21, 22^, while the host provides nutrients as well as protection against *Chlorella* viruses that are abundant in nature and otherwise capable of infecting the endosymbionts^23, 24^. The relationship is also coordinated because the host cell cycle can regulate endosymbiont division^25, 26^. In many cases, the *Chlorella* endosymbionts can be cultured under laboratory conditions outside of the host; however, the growth media requires the addition of nutrients and organic nitrogen sources such as amino acids or urea, which are usually provided by the hosts^27–29^. The altered metabolism, coordinated cell division and increased viral susceptibility suggests that these endosymbionts are unlikely to be viable in nature as free-living species, and it implies that the endosymbiotic relationship may be in the early stages of transition from facultative to obligate.

Although genomic data is accumulating at a rapid rate in Trebouxiophyceae, few studies have focused on the genomic effects of the evolutionary transition to endosymbiosis in this group. At the nuclear level, the genome of the endosymbiont *Chlorella variabilis* NC64A is 46.6 Mb in size with 9,791 annotated genes, which is about 20% smaller with 5% fewer genes than the free-living species *Chlorella pyrenoidosa* ^30, 31^. Without more *Chlorella* nuclear genomes, however, it cannot be determined whether the smaller genome in *C. variabilis* NC64A results from a transition to endosymbiosis. More distantly related species within Chlorellaceae have a wide range of nuclear genome sizes, from 48.8 Mb in *Coccomyxa subellipsoidea* C-169 to only 13.3 Mb in *Picochlorum* sp. and 22.9 Mb in *Auxenochlorella protothecoides*, with the latter two genomes postulated to be the result of large-scale reduction unrelated to endosymbiotic transitions^32–34^.

In contrast to the relatively limited number of nuclear genomes, there are presently more than 30 plastomes and 10 mitogenomes from Trebouxiophyceae that have been fully sequenced, including multiple representatives of free-living *Chlorella* species. The plastomes are highly variable, ranging in size from 37 kb to >300 kb and containing 54–114 genes, while the mitogenomes are more conserved, varying from 49 kb to 85 kb in size with 56–62 genes^15, 35–37^. Importantly, plastomes and mitogenomes of multiple free-living *Chlorella* species are available, providing the necessary evolutionary context to examine the organellar genomic effects of a transition to an endosymbiotic lifestyle in this genus. To explore this issue, complete plastomes and mitogenomes were sequenced from three endosymbiotic green algae: *Chlorella heliozoae* SAG 3.83 (isolated from *Acanthocystis turfacea*), *Chlorella variabilis* Syngen 2-3 (isolated from *P. bursaria*) and *Micractinium conductrix* strain Pbi (isolated from *P. bursaria*).

## Results

### Comparative analysis of Chlorellaceae mitogenomes

The three newly sequenced mitogenomes from the endosymbiotic green algae *C. heliozoae*, *C. variabilis* Syngen and *M. conductrix* were circular assemblies and 62,477 bp, 79,601 bp and 74,708 bp in length, respectively (Figure S1). Depth of coverage analysis showed that the genomes are deeply (>500x) and evenly covered (Figure S2), providing support for the accuracy of the assembly. A broader comparison of mitogenomes from species in Trebouxiophyceae showed that genome size varies from 49 kb to 79 kb (Table 1). Notably, the four endosymbionts (*C. heliozoae*, *C. variabilis* Syngen, *C. variabilis* NC64A and *M. conductrix*) tend to have relatively larger mitogenome sizes compared with free-living individuals (*A. protothecoides*, *Chlorella* sp. ArM0029B, *Chlorella sorokiniana*, *C*. *subellipsoidea* C-169, *Lobosphaera incisa* and Trebouxiophyceae sp. strain MX-AZ01) and substantially larger mitogenome sizes compared with parasitic, nonphotosynthetic green algae (*Helicosporidium* sp. and *Prototheca wickerhamii*). Despite the wide range of sizes, it is noteworthy that the Trebouxiophyceae mitogenomes carry very similar gene content (Table 1). In fact, the mitogenomes from all five *Chlorella* species and *M. conductrix* share the same set of 32 protein-coding genes, three rRNAs, and 27 tRNAs, indicating that mitochondrial gene content is not affected by the different lifestyles among these species.

**Table 1:** Comparison of general features among mitogenomes and plastomes ﻿of selected Trebouxiophyceae.

	Mitochondrial Genome						Plastid Genome					
	Size (bp)	AT%	Protein	tRNA	rRNA	Intron	Size (bp)	AT%	Protein	tRNA	rRNA	Intron
**Endosymbionts**												
*Chlorella heliozoae* SAG 3.83	62477	68.3	32	27	3	7	124353	64.7	79	31	3	2
*Chlorella variabilis* Syngen 2-3	79601	71.9	32	27	3	7	124881	66.1	79	32	3	3
*Chlorella variabilis* NC64A	78500	71.8	32	27	3	6	124793	66.0	79	32	3	3
*Micractinium conductrix* Pbi	74708	70.6	32	27	3	5	129436	65.2	79	32	3	10
**Free-living algae**												
*Auxenochlorella protothecoides*	57274	71.3	32	26	3	7	84576	69.2	76	30	3	0
*Chlorella* sp. ArM0029B	65049	71.5	32	27	3	1	119989	66.1	79	32	3	1
*Chlorella sorokiniana*	52528	70.9	32	27	3	1	109803	66.0	78	31	3	2
*Coccomyxa subellipsoidea* C-169	65497	46.8	30	26	3	5	175731	49.3	79	33	3	1
*Lobosphaera incisa*	69997	64.0	32	24	3	4	156028	72.1	78	30	3	1
*Marvania geminata*	—	—	—	—	—	—	108470	61.8	78	31	3	0
Trebouxiophyceae sp. MX-AZ01	74423	46.6	30	23	3	11	149707	42.3	79	33	3	5
**Parasites**												
*Helicosporidium* sp.	49343	74.4	32	25	3	4	37454	73.1	26	25	3	1
*Prototheca wickerhamii*	55328	74.2	30	26	3	5	55636	68.8	40	28	3	1

**Figure 1:**
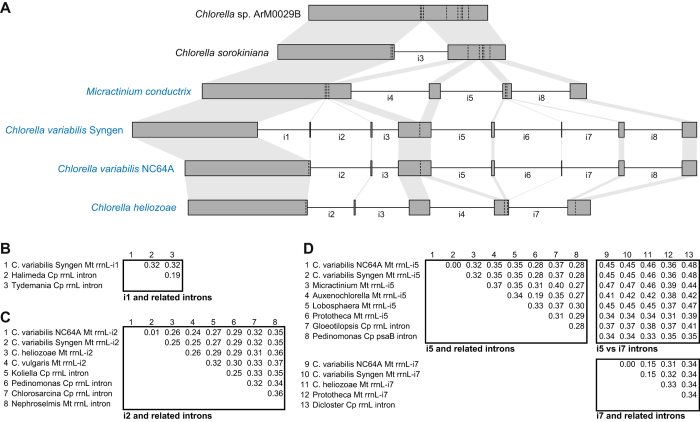
(**A**) Comparison of intron content in the mitochondrial *rrnL* gene. Exons are marked by dark gray rectangular boxes, and regions of exon homology among species are highlighted by light gray shading. Eight different introns (labeled i1–i8) are sporadically distributed in the *rrnL* gene among Chlorellaceae species. Introns when present are denoted by horizontal black lines and named by their relative position within the gene. The location of introns that are absent from the *rrnL* gene are marked by a vertical dotted line. Species names are shown on the left, and endosymbionts are shown in blue text. The maps are drawn approximately to scale. (**B**) Pairwise distances of intron *rrnL* i1 and homologs. (**C**) Pairwise distances of intron *rrnL* i2 and homologs. (**D**) Pairwise distances of introns *rrnL* i5 and i7 and their homologs. Sequence alignments for all pairwise distance calculations are shown in Supplementary Figure S3.

In contrast to the stable gene content, introns are highly variable among Trebouxiophyceae mitogenomes (Table 2), ranging from a minimum of one intron (*C*. sp. ArM0029B and *C. sorokiniana*) to a maximum of 11 introns (Trebouxiophyceae sp.). The gene for the large ribosomal RNA subunit (*rrnL*) contains the most introns and exhibits substantial variation in content among Chlorellaceae species: the free-living *C*. sp. ArM0029B does not have any introns and the free-living *C. sorokiniana* has only one, while the four endosymbiotic species have 3–7 introns (Fig. 1A). Targeted RT-PCR and cDNA sequencing of *C. heliozoae*, *C. sorokiniana*, *C. variabilis* Syngen and *M. conductrix* confirmed that these variable regions in *rrnL* are truly introns that are removed from the mature transcript. To assess the possible origin of these Chlorellaceae introns, homologs were identified using blast followed by sequence alignment (Figure S3) and pairwise distance (*p*) calculations (Fig. 1B–D). Intron 1 of *rrnL*, which is present in *C. variabilis* Syngen, exhibits strong similarity (e-value < 1e^−20^) to plastid introns from two green algal species (*Halimeda discoidea* and *Tydemania expeditionis*) in the Bryopsidales (Fig. 1B; Figure S3A). The *rrnL*-i2 intron is present in several *Chlorella* species and is highly similar to plastid *rrnL* introns from many green algal species in diverse lineages such as Chlorophyceae (*Chlorosarcina brevispinosa*), Pedinophyceae (*Pedinomonas tuberculata*), and Streptophyta (*Koliella corcontica*), and slightly less similar to only one mitochondrial intron from the prasinophyte *Nephroselmis olivacea* (Fig. 1C; Figure S3B). Introns 5 and 7 have closest homologs to plastid introns, but they also exhibit some weaker similarity to one another (Fig. 1D; Figure S3C). Most other Chlorellaceae mitochondrial introns have only weak similarity to other introns available in GenBank, limiting any inferences about their origins.

**Table 2:** Comparison of intron content in Trebouxiophyceae mitogenomes and plastomes.

	Mitogenome									Plastome										
	*cob*	*cox1*	*rrnL*	*trnH*	trnS*	*trnW*	*trnC*	*trnK*	*trnF*	*chlL*	*ftsh*	*petB*	*psaC*	*psbA*	*psbB*	*psbC*	*psbD*	*rps12*	*rrnL*	*trnL*
**Endosymbionts**																				
*Chlorella heliozoae* SAG 3.83	1	2	4	0	0	0	0	0	0	0	0	0	0	0	0	1	0	0	1	0
*Chlorella variabilis* Syngen 2-3	0	0	7	0	0	0	0	0	0	0	0	0	0	1	0	1	0	0	0	1
*Chlorella variabilis* NC64A	0	0	6	0	0	0	0	0	0	0	0	0	0	1	0	1	0	0	0	1
*Micractinium conductrix* Pbi	0	2	3	0	0	0	0	0	0	0	0	1	1	0	1	0	1	1	4	1
**Free-living algae**																				
*Auxenochlorella protothecoides*	0	3	4	0	0	0	0	0	0	0	0	0	0	0	0	0	0	0	0	0
*Chlorella* sp. ArM0029B	0	1	0	0	0	0	0	0	0	0	0	0	0	0	0	0	0	0	0	1
*Chlorella sorokiniana*	0	0	1	0	0	0	0	0	0	1	0	0	0	0	0	0	0	0	0	1
*Coccomyxa subellipsoidea* C-169	0	0	1	1	2	1	0	0	0	0	0	0	0	0	1	0	0	0	0	0
*Lobosphaera incisa*	0	0	1	0	0	0	1	1	1	0	×	0	0	0	0	0	0	0	0	1
*Marvania geminata*	—	—	—	—	—	—	—	—	—	0	0	0	0	0	0	0	0	0	0	0
Trebouxiophyceae sp. MX-AZ01	0	1	6	1	2	1	0	0	0	0	1	0	0	1	0	0	0	0	3	0
**Parasites**																				
*Helicosporidium* sp.	0	2	2	0	0	0	0	0	0	×	0	0	0	0	0	0	0	0	0	1
*Prototheca wickerhamii*	0	3	2	0	0	0	0	0	0	×	0	0	0	0	0	0	0	0	0	1

^*^There are two copies of this gene with one intron each.  × Gene loss.

In terms of adenine and thymine (AT) content, there is no obvious correlation with an endosymbiont lifestyle in *Chlorella*. All three newly sequenced endosymbionts are AT rich at ~70%, which is similar to that of the endosymbiont *C. variabilis* NC64A (71.8%) as well as the free-living species *A. protothecoides* (71.3%), *C*. sp. ArM0029B (71.5%) and *C. sorokiniana* (70.9%). In contrast, AT content is higher in the two nonphotosynthetic parasites *Helicosporidium* sp. (74.4%) and *P. wickerhamii* (74.2%), but much lower in the other free-living trebouxiophytes *C. subellipsoidea* C-169 (46.9%), *L. incisa* (64.0%) and Trebouxiophyceae sp. (46.6%) (Table 1).

### Comparative analysis of Chlorellaceae plastomes

The plastomes from *C. heliozoae*, *C. variabilis* Syngen and *M. conductrix* have circularly mapping structures, with lengths of 124,353 bp, 124,881 bp, and 129,436 bp, respectively (Figure S4). The genomes exhibit deep and even coverage (Figure S5), consistent with an accurate assembly. All three newly sequenced plastomes harbor the same 79 protein-coding genes, which are also mostly or completely present in the other photosynthetic trebouxiophytes (Table 1). A comparison of intron content among species indicated substantial variation: the *M. conductrix* plastome is rich with 10 introns, whereas other taxa in Chlorellaceae contain 0–3 introns (Table 2). BLAST homology searches of Chlorellaceae plastid introns identified many good matches (e-value < 1e^−20^) with plastid introns from other green algae. The AT content is highly conserved in *Chlorella* (Table 1), in which the free-living *C*. sp. ArM0029B and *C. sorokiniana*, (66.1% and 66.0% in AT content, respectively) have AT contents that are very close to the endosymbiotic Chlorellaceae lineages (64.7–66.1%).

In comparison with other selected Trebouxiophyceae with various lifestyles, there is no indication of a reduction in genome size or gene content in the *Chlorella* endosymbionts, nor any strong correlation with lifestyle and AT content or intron content. In contrast, the nonphotosynthetic parasitic lineages *Helicosporidium* sp. and *P. wickerhamii* exhibit a major reduction in genome size, significant gene loss, and the highest AT content, which is likely associated with the transition to parasitism and accompanying loss of photosynthetic ability^15, 38^.

### Phylogenetic analysis

**Figure 2:**
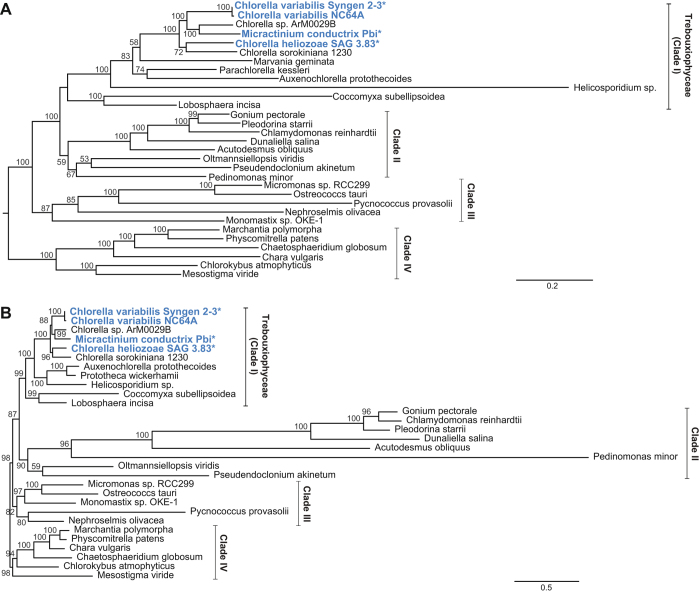
Phylogenetic analysis of selected trebouxiophytes by (**A**) 74 plastid genes and (**B**) 32 mitochondrial genes. The trees shown were generated by maximum likelihood and numbers indicating at each node represent bootstrap values. Weak support values (<50%) were eliminated from the figure. Endosymbiont species in Chlorellaceae are highlighted in bold blue text. The three species that were newly sequenced in this study are marked with an asterisk. Trees were rooted on streptophytes. The scale bars were shown at the bottom right for each tree.

To assess the relationships among the endosymbiont and free-living species of Chlorellaceae, we performed ML phylogenetic analyses with the GTR + G + I model using data sets of 32 mitochondrial genes or 74 plastid genes from the three new endosymbionts plus a diverse selection of other green algae for which complete mitogenomes and plastomes are available (Table S1). Overall, the two phylogenies are highly consistent (Fig. 2), with four major clades: the first clade includes the Trebouxiophyceae; the second clade includes Chlorophyceae, Ulvophyceae, and Pedinophyceae; the third clade includes Mamiellophyceae, Nephroselmidophyceae, and Prasinophyceae; and the final clade includes the outgroup Streptophyta. Notably, *M. conductrix* is nested within the *Chlorella* clade of species with maximal bootstrap support (100%) in both the plastid and mitochondrial phylogenies, suggesting that taxonomic reassessment of *Chlorella* and *Micractinium* is warranted.

Comparison of the distribution of endosymbionts within the Trebouxiophyceae indicated that the endosymbiont lifestyle has evolved multiple times in *Chlorella* (Fig. 2). The common ancestor of the four endosymbionts and two free-living *Chlorella* species received maximal support in both ML analyses, as reflected by the bootstrap value of 100%. In both trees, the free-living *C*. sp. ArM0029B was positioned as sister to the endosymbiotic *M. conductrix* with strong bootstrap support (>99%). Additionally, the free-living *C. sorokiniana* was sister to the endosymbiotic *C. heliozoae* with good bootstrap support (72–96%). The two endosymbionts *C. variabilis* NC64A and *C. variabilis* Syngen grouped very strongly and showed little divergence, confirming a close relationship between these taxa. Importantly, the four endosymbiont species do not group together in a single clade, implying that the endosymbiont lifestyle has evolved more than once in *Chlorella*. Alternative topologies constraining the endosymbiotic lineages to a single clade forming a monophyletic group were strongly rejected by the Shimodaira-Hasegawa (SH) Test (P < 0.01). These results are consistent with the hypothesis of multiple evolutionary origins of endosymbiosis within Chlorellaceae, as also reported in previous studies^19, 39–41^.

## Discussion

### A lack of genomic reduction in the organellar genomes of Chlorellaceae endosymbionts

Although it is well established that endosymbiont genomes in obligate endosymbioses experience massive reduction, less is known about the timing of genomic reduction relative to the transition from a free-living species or facultative endosymbiont to an obligate endosymbiont. In this study, we determined the complete mitogenomes and plastomes from three endosymbiotic algae (*C. heliozoae*, *C. variabilis* Syngen and *M. conductrix*) whose altered biology and increased viral susceptibility suggest that they are in the early stages of transition from a facultative to an obligate endosymbiotic lifestyle. Using these new organellar genomes, and those previously available from the endosymbiont *C. variabilis* NC64A, we examined the genomic effects of the evolutionary transition from a facultative to an obligate endosymbiotic lifestyle within the Trebouxiophyceae. In the mitogenome, there is a very similar set of protein-coding genes within the various trebouxiophytes with different lifestyles (Table 1). This is perhaps not unexpected since the mitochondrial genes are essential for respiration, and this metabolic process is required for algae of all lifestyles.

As to the plastome, the newly sequenced green algal species have a normal gene repertoire with 79 protein genes (Table 1), which is the same or slightly more than in seven free-living trebouxiophyte species (*A. protothecoides*, *C*. sp. ArM0029B, *C. sorokiniana, C. subellipsoidea* C-169, *L. incisa*, *Marvania geminata* and Trebouxiophyceae sp. MX-AZ01) and another endosymbiotic *Chlorella* species (*C. variabilis* NC64A), but differs markedly from the two heterotrophic algae (*Helicosporidium* sp. and *P. wickerhamii*) whose parasitic nature has resulted in the loss of all photosynthesis-related genes^15, 38^. The loss of plastid-encoded photosynthesis-related genes or even the complete plastome has also been documented many times in algae such as *Cryptomonas paramecium* and *Polytomella* ^42, 43^. These examples suggest that the loss of photosynthesis causes the loss of most or all of the photosynthetic genes, leading to dramatic changes in plastomes. Conversely, our three endosymbiont species appear to need the ability to create energy for themselves or for their hosts by photosynthesis. This ability also allows them to grow outside of their host cells under laboratory conditions, although they need additional resources, such as organic nitrogen sources and vitamins, in order to survive^27–29^.

Overall, our results do not indicate any organellar genome reduction in endosymbiotic green algae, in contrast to the reduction in genome content observed in facultative endosymbiotic bacteria^11–14^. It is possible that organellar genomes only decrease in size once the endosymbiotic relationship becomes fully obligatory for both endosymbiont and host, or if the relationship shifts from mutualistic to parasitic, as in parasitic land plants and parasitic algae^15, 38, 44–46^. Further investigation of green algae with fully established obligate endosymbiotic relationships are needed to determine if the endosymbiotic lifestyle results in genomic reduction of organellar genomes.

### Major variation in intron content among Trebouxiophyceae organellar genomes

In the mitogenome of Trebouxiophyceae species, two protein-coding genes (*cob* and *cox1*), the *rrnL* ribosomal RNA gene and six tRNAs are interrupted by introns (Table 2). Notably, the endosymbiotic lineages generally have more introns than the free-living taxa. In the plastome, the presence of introns in the Trebouxiophyceae species is highly sporadic and lineage specific, with relatively few introns in most species that are scattered among 10 protein genes as well as *rrnL* and *trnL* (Table 2). Like the mitogenomes, the plastomes of the endosymbiotic species tend to be more intron rich, possessing 10 introns in *M. conductrix*, three in *C. variabilis* Syngen and *C. variabilis* NC64A, and two in *C. heliozoae*. These findings are potentially intriguing because they would be consistent with horizontal transfer of organellar introns among endosymbionts or with the host. However, while this pattern is intriguing, there is too little data at this stage to infer much from this apparent trend. Additional data from more endosymbionts and free-living Chlorellaceae are needed.

The sporadic distribution of these organellar introns in Trebouxiophyceae raises questions about their origin and subsequent evolution. Were these introns acquired horizontally, which has been postulated for several introns in other plant lineages^47, 48^? Were they propagated to ectopic sites within the same genome, as reported for a few organellar introns^48–50^? Or were they transferred vertically from a highly intron-rich common ancestor, followed by extensive loss of many introns from the descendent lineages? For *rrnL* introns i1, i2, i5, and i7, they show strong similarity to plastid introns from distantly related species, suggesting a possible horizontal origin (Fig. 2B–D; Figure S3). Interestingly, introns i5 and i7 show homology with one another, which suggests that one of these introns originated by intragenomic propagation of the other intron (Figure S3C). For most of the remaining introns, the lack of strong matches to any other introns available in GenBank limits any inferences about their possible origin. A more extensive analysis of intron content among trebouxiophycean green algae should help resolve this issue.

### Endosymbionts evolved multiple times within Chlorellaceae

The endosymbiotic lifestyle has evolved many times in green algae, as indicated by the multiple independent clades of endosymbiont algae in phylogenetic trees based on the nuclear ITS and 18S rRNA sequences^19^. Even within the Chlorellaceae, endosymbionts do not cluster together in molecular phylogenies, suggesting that this lifestyle evolved multiple times^19, 39–41^. However, a major limitation in these previous studies was the small data sets used to construct the trees, which have been based on nuclear rRNA data sets totaling ~2,000 bp.

In our study, phylogenies inferred from the plastid (74 genes) and mitochondrial (32 genes) data sets indicated that the endosymbionts among Chlorellaceae species do not cluster together, providing strong evidence for independent transitions to endosymbiosis (Fig. 2). Furthermore, these endosymbionts serve as specific hosts for large dsDNA viruses known as chloroviruses^23, 28^, and the high degree of specificity of the viruses for a particular endosymbiont clade provides further evidence for the evolutionary distinction among the endosymbionts^51^. Collectively, the data from all three plant genomes provides clear evidence that the endosymbiotic lifestyle arose multiple times in Chlorellaceae. Similarly, some algal symbionts in lichens were shown to be closely related, yet these lichen endosymbioses still appear to have independent origins^52, 53^. Our results of multiple transitions to symbiosis is also comparable with the multiple transitions to parasitism that have occurred in flowering plants and red algae^54, 55^.

### Taxonomic treatment of Micractinium and Chlorella

The taxonomic treatment of *Chlorella* and related organisms has had a complicated history, owing primarily to the limited morphological diversity among species traditionally grouped within this genus, while molecular analyses have resulted in a major revision of the species that are true relatives of the type species *Chlorella vulgaris* and revealed a surprising morphological diversity among these relatives^19, 41, 56^. As part of this taxonomic revision, the emerging consensus of three distinct endosymbiont clades within Chlorellaceae has led to their classification into three species: *C. heliozoae* (for SAG 3.83-related endosymbionts of *A. turfacea*), *C. variabilis* (for NC64A-related endosymbionts of *P. bursaria*), and either *M. conductrix or Micractinium reisseri* (for Pbi-related endosymbionts of *P. bursaria*)^19, 41, 57^. Although the various authors have disagreed on the correct specific epithet of *conductrix* or *reisseri* for the Pbi-related endosymbionts^19, 57^, there is general agreement that these endosymbionts should be classified into a separate genus, *Micractinium*, based on the 18S and ITS data sets^19, 41, 56^.

Our results, however, based on the phylogenomic analyses inferred from mitochondrial and plastid genes, indicate that this Pbi endosymbiont clusters clearly within the genus *Chlorella*, grouping specifically as the sister taxon to *C*. sp. ArM0029B (Fig. 2). These phylogenomic results, supported by robust organellar genomic phylogenies, call into question the placement of this endosymbiont into a separate genus, suggesting instead that it should be classified as a species of *Chlorella*. Alternatively, it is possible that the genus name *Chlorella* is polyphyletic, arguing for a recircumscription of this genus and the reclassification of some species into new genera. Another possibility is that there may be hard incongruence between nuclear and organellar loci due to independent evolutionary histories of the different genomes. Sequencing of complete organellar genomes from additional Chlorellaceae species, particularly from other *Micractinium* taxa, will be critical in resolving this issue. Sampling of additional nuclear loci, other than the nuclear rRNA and ITS regions, will also be crucial to address the apparently conflicting signals between the nuclear and organellar genomes.

## Materials and Methods

### Source of species

The Van Etten lab obtained isolates of *Chlorella heliozoae* (SAG 3.83) in 2006*, Chlorella variabilis* Syngen 2-3 (ATCC 30562) in 2012, and *Micractinium conductrix* (Pbi) in 1986, and these isolates have been subsequently maintained in continuous cell culture at the University of Nebraska–Lincoln. For this study, *C. heliozoae* and *C. variabilis* Syngen liquid cell cultures were grown at room temperature in Modified Bold’s Basal Medium^58^ and *M. conductrix* was grown on FES medium^59^ according to established procedures^24^. Cells were pelleted by centrifugation at 3000 × g for 10 minutes. All cells were stored at −80 °C prior to DNA extraction.

### DNA extraction and genome sequencing

DNA extraction was performed using a modified preparation protocol^60, 61^. About 0.2 g of the harvested cells were resuspended in 375 µL SDS-EB buffer (2% SDS; 100 mM Tris-HCl, pH 8.0; 400 mM NaCl; 40 mM EDTA, pH 8.0), and then an equal volume of water was added, followed by 750 µL phenol:chloroform:isoamyl alcohol (25:24:1). To break the cell wall, 25 mg of glass beads (425–600 µm) were added in the same tube, and the sample was vortexed for 5 min. Cellular debris was pelleted by centrifuging for 5 min at 12,000 × g. The aqueous solution was transferred to a new tube, and 2 µL RNase (10 mM) was added, followed by 30 min incubation at 37 °C. The phenol:chloroform:isoamyl alcohol extraction and centrifugation steps were repeated without glass beads, and the aqueous solution was then treated with 750 µL chloroform. The supernatant was transferred to a new tube, and then twice the volume of 100% ethanol was added and incubated for 1 hr at −20 °C. DNA was collected by centrifugation for 20 min at 12,000 × g followed by washing with 70% ethanol. The DNA was air dried and then resuspended in 50 µL water.

The DNA samples were sent to the High-Throughput DNA Sequencing and Genotyping Core Facility at the University of Nebraska Medical Center (Omaha, NE). For each sample, 20–30 M reads of 100 bp were sequenced from a 500 bp paired-end library on an Illumina HiSeq 2500.

### Genome assembly and annotation

The organellar genomes of *C. heliozoae*, *C. variabilis* Syngen and *M. conductrix* were assembled from the Illumina sequence reads by running Velvet version 1.2.03^62^ using different pairwise combinations of Kmer (61, 71, 81, 91) and expected coverage (50, 100, 200, 500, 1000) values, as described previously^63, 64^. Scaffolding was turned off and the minimum coverage parameter was set to 10% of expected coverage. To verify the species identities of the three endosymbionts, the sequence for the intact nuclear rRNA region (including 18S rRNA, ITS1, 5.8S rRNA, ITS2, and 26S rRNA) was extracted from one of the assemblies for each species and used in a blast analysis against the NCBI nucleotide database to find the closest sequence match. The nuclear rRNA region assembled from our sequenced samples (*C. heliozoae* SAG 3.83, *C. variabilis* Syngen 2-3, and *M. conductrix* Pbi) were each 99.97% identical, respectively, to accession numbers FM205850 from *Chlorella* SAG 3.83 (3851/3852 sites), AB206550 from *C. variabilis* Syngen 2-3 (3947/3848 sites), and AB506070 from *M. reisseri* (6457/6459 sites). The near 100% sequence identity of our assembled sequences to the same species in GenBank provides strong verification of the organismal identity of our sampled species.

For each assembly, plastid and mitochondrial contigs were detected by blastn searches with known organellar gene sequences from related Chlorellaceae species used as queries. The final consensus sequence for each species was constructed by aligning the mitochondrial and plastid contigs from the best draft assemblies (that maximized the average length of plastid or mitochondrial contigs). Circular genomes were confirmed by aligning the overlapping terminal regions of the contigs, which was further supported by read pairs that spanned both ends of the assembly. Using this strategy, a single completed circular chromosome was assembled for the plastome and mitogenome of each species.

To evaluate the depth of coverage of the genome assemblies, read pairs were mapped onto respective consensus sequences with Bowtie 2.0^65^. The resulting plots show an average mitochondrial depth of coverage of approximately 5000x for *C. heliozoae*, 4500x for *C. variabilis* Syngen, and 500x for *M. conductrix* (Figure S2), and an average plastid depth of coverage of roughly 8000x for *C. heliozoae*, 3500x for *C. variabilis* Syngen, and 300x for *M. conductrix* (Figure S5). The depths of coverage for the organellar genomes are substantially higher than would be expected for the nuclear genome from these small sequenced data sets, such that any organellar sequence copies in the nuclear genome will not contribute to the constructed sequences of the organellar genomes. In addition, there are no regions of substantially lower coverage in the organellar coverage plots, arguing against any erroneous incorporation of nuclear DNA into the organellar genome assemblies.

Mitochondrial protein-coding genes were annotated by blast against the non-redundant database from the National Center for Biotechnology Information. The protein genes from the plastome were initially annotated by using DOGMA^66^ with a 60% cutoff and a blast e-value of 1e^−5^, followed by manual adjustment as necessary. Ribosomal RNAs were identified by blastn searches^15^ and transfer RNAs were identified with tRNAscan-SE^67^. To identify potentially novel genes, blastn and blastx searches were also applied to all noncoding regions but no additional genes were identified. Homologs to mitochondrial and plastid introns in Chlorellaceae were identified by a blastn search with an e-value cutoff of 1e^−20^. Homologous introns were aligned in MEGA version 7^68^ using the MUSCLE algorithm, and then uncorrected *p*-distances were calculated in MEGA using the distance function with deletions removed in a pairwise manner.

### RNA extraction and cDNA sequencing

To verify intron content in the mitochondrial large ribosomal RNA (*rrnL*), cDNA was amplified and sequenced for *C. heliozoae*, *C. sorokiniana*, *C. variabilis* Syngen and *M. conductrix*. Total RNA was isolated from ~1 × 10^9^ cells by using the following modified Trizol protocol. Harvested cells were spread on the wall of the Eppendorf tube before freezing in liquid nitrogen for 1 min, then 3 ml of Trizol was immediately added to the frozen pellet and the tube was vortexed for 10 min. The homogenized sample was incubated for another 5 min at room temperature followed by centrifugation at 12,000 × g for 10 min at 4 °C to remove insoluble material and polysaccharides. After transferring the supernatant to a new tube, 0.75 ml of chloroform was added with vigorous shaking for 30 sec, and the tube was vortexed for 2 min. The sample was incubated for 5 min at room temperature followed by centrifugation at 12,000 × g for 15 min at 4 °C. The aqueous phase was transferred to a clean tube, and then an equal volume of phenol:chloroform:isoamyl alcohol (25:24:1) was added and the sample was vortexed for 2 min. The centrifugation process was repeated, and the aqueous phase of the sample was transferred to a new tube and an equal volume of isopropanol was added to the solution, followed by incubation at −20 °C for 30 min. The RNA was collected by centrifugation, washed, air dried and dissolved in 20 µL RNase-free water following the Trizol reagent user guide.

With the isolated RNA as template, RT-PCR and cDNA sequencing were carried out by the approaches described previously^69^. Species-specific primers were designed for various regions of *rrnL* to amplify the total length of this cDNA. The PCR-amplified *rrnL* cDNAs were Sanger sequenced on both strands at GenScript (Piscataway, NJ).

### Phylogenetic analysis

Both plastid and mitochondrial phylogenies were generated in this study. In addition to the three newly sequenced species, organellar genomes from 24–25 representative chlorophytes and six streptophytes (Table S1) were collected from GenBank. Individual protein-coding genes were extracted and then manually checked for any misannotation issues. Exons from all 74 plastid genes and 32 mitochondrial genes that were present in more than half of the taxa were aligned by codons using MUSCLE version 3.8.31^70^, and manually adjusted in BioEdit version 7.2.0 if necessary. Introns were not analyzed phylogenetically due to their highly sporadic distribution among species. Plastid and mitochondrial protein gene data sets were concatenated seperately by FASconCAT version 1.0^71^, generating 124,056 and 38,679 aligned sites, respectively. The ambiguously aligned regions in the concatenated alignments were excluded using Gblocks version 0.91b^72^ with relaxed parameters (t = c, b2 = 16, b4 = 5, b5 = half), retaining 49,863 sites (40%) of the original plastid alignment and 19,965 sites (51%) of the original mitochondrial alignment. The nucleotide substitution model of best fit was determined to be the GTR + G + I model by jModelTest 2.1.10^73^. Phylogenetic analyses were inferred from plastid and mitochondrial data sets using the Maximum Likelihood (ML) approach in PhyML version 3.0^74^. ML trees were estimated with the GTR + G + I model and confidence of branching was estimated by bootstrap analyses with 100 replicates.

## Electronic supplementary material

Supplementary Information
