# Supplementary material for: Multiple origins of endosymbionts in Chlorellaceae with no reductive effects on the plastid or mitochondrial genomes
Source: Sci Rep. 2017 Aug 30;7:10101. doi: 10.1038/s41598-017-10388-w (PMC5577192; doi:10.1038/s41598-017-10388-w)

Multiple origins of endosymbionts in Chlorellaceae with no reductive effects on the plastid or mitochondrial genomes

Weishu Fan[1, 2], Wenhu Guo[3], James L. Van Etten[4], Jeffrey P. Mower[1, 2]

[1] Center for Plant Science Innovation, University of Nebraska, Lincoln, NE 68588, USA

[2] Department of Agronomy and Horticulture, University of Nebraska, Lincoln, NE 68583, USA

[3] Wuhan Frasergen Bioinformatics Co. Ltd., Wuhan, Hubei 430075, China

[4] Department of Plant Pathology and Nebraska Center for Virology, University of Nebraska, Lincoln, NE 68583, USA

Table S1. GenBank accession numbers for taxa used in this study

Group	Family	Species	Accession number	
			**Plastome**	**Mitogenome**
Chlorophyceae	Chlamydomonadaceae	*Chlamydomonas reinhardtii*	NC_005353	NC_001638
Chlorophyceae	Dunaliellaceae	*Dunaliella salina*	NC_016732	NC_012930
Chlorophyceae	Scenedesmaceae	*Acutodesmus obliquus*	NC_008101	NC_002254
Chlorophyceae	Volvocaceae	*Gonium pectorale*	NC_020438	NC_020437
Chlorophyceae	Volvocaceae	*Pleodorina starrii*	NC_021109	NC_021108
Mamiellophyceae	Bathycoccaceae	*Ostreococcus tauri*	NC_008289	NC_008290
Mamiellophyceae	Mamiellaceae	*Micromonas* sp. RCC299	NC_012575	NC_012643
Mamiellophyceae	Monomastigaceae	*Monomastix* sp. OKE-1	NC_012101	NC_022797
Pedinophyceae	Pedinomonadaceae	*Pedinomonas minor*	NC_016733	NC_000892
Prasinophyceae	Pycnococcaceae	*Nephroselmis olivacea*	NC_000927	NC_008239
Prasinophyceae	Pycnococcaceae	*Pycnococcus provasolii*	NC_012097	NC_013935
Streptophyta	Chaetosphaeridiaceae	*Chaetosphaeridium globosum*	NC_004115	NC_004118
Streptophyta	Characeae	*Chara vulgaris*	NC_008097	NC_005255
Streptophyta	Chlorokybaceae	*Chlorokybus atmophyticus*	NC_008822	NC_009630
Streptophyta	Funariaceae	*Physcomitrella patens*	AP005672	NC_007945
Streptophyta	Marchantiaceae	*Marchantia polymorpha*	NC_001319	NC_001660
Streptophyta	Mesostigmataceae	*Mesostigma viride*	NC_002186	NC_008240
Trebouxiophyceae	Chlorellaceae	*Auxenochlorella protothecoides*	NC_023775	KC843974
Trebouxiophyceae	Chlorellaceae	*Chlorella* sp. ArM0029B	KF554427	KF554428
Trebouxiophyceae	Chlorellaceae	*Chlorella variabilis* Syngen	KY629617	KY629618
Trebouxiophyceae	Chlorellaceae	*Chlorella heliozoae*	KY629616	KY629615
Trebouxiophyceae	Chlorellaceae	*Chlorella sorokiniana*	NC_023835	KM241869
Trebouxiophyceae	Chlorellaceae	*Chlorella variabilis* NC64A	KJ718922	NC_025413
Trebouxiophyceae	Chlorellaceae	*Helicosporidium sp.*	NC_008100	NC_017841
Trebouxiophyceae	Chlorellaceae	*Lobosphaera incisa*	KM821265	NC_027060
Trebouxiophyceae	Chlorellaceae	*Marvania geminata*	NC_025549	n/a
Trebouxiophyceae	Chlorellaceae	*Micractinium conductrix*	KY629620	KY629619
Trebouxiophyceae	Chlorellaceae	*Parachlorella kessleri*	NC_012978	n/a
Trebouxiophyceae	Chlorellaceae	*Prototheca wickerhamii*	KJ001761	NC_001613
Trebouxiophyceae	Coccomyxaceae	*Coccomyxa subellipsoidea*	NC_015084	NC_015316
Ulvophyceae	Kornmanniaceae	*Pseudendoclonium akinetum*	NC_008114	NC_005926
Ulvophyceae	Oltmannsiellopsidaceae	*Oltmannsiellopsis viridis*	NC_008099	NC_008256

Figure S1. Mitogenome maps of *C. heliozoae*, *C. variabilis* Syngen and *M. conductrix*. Outer genes are transcribed counter-clockwise; inner genes are transcribed clockwise. Gene and intron colors correspond to the functional categories listed in the key at the top left. GC content is shown on the inner circle by dark grey bars. The map was drawn with OgDraw (http://ogdraw.mpimp-golm.mpg.de/).

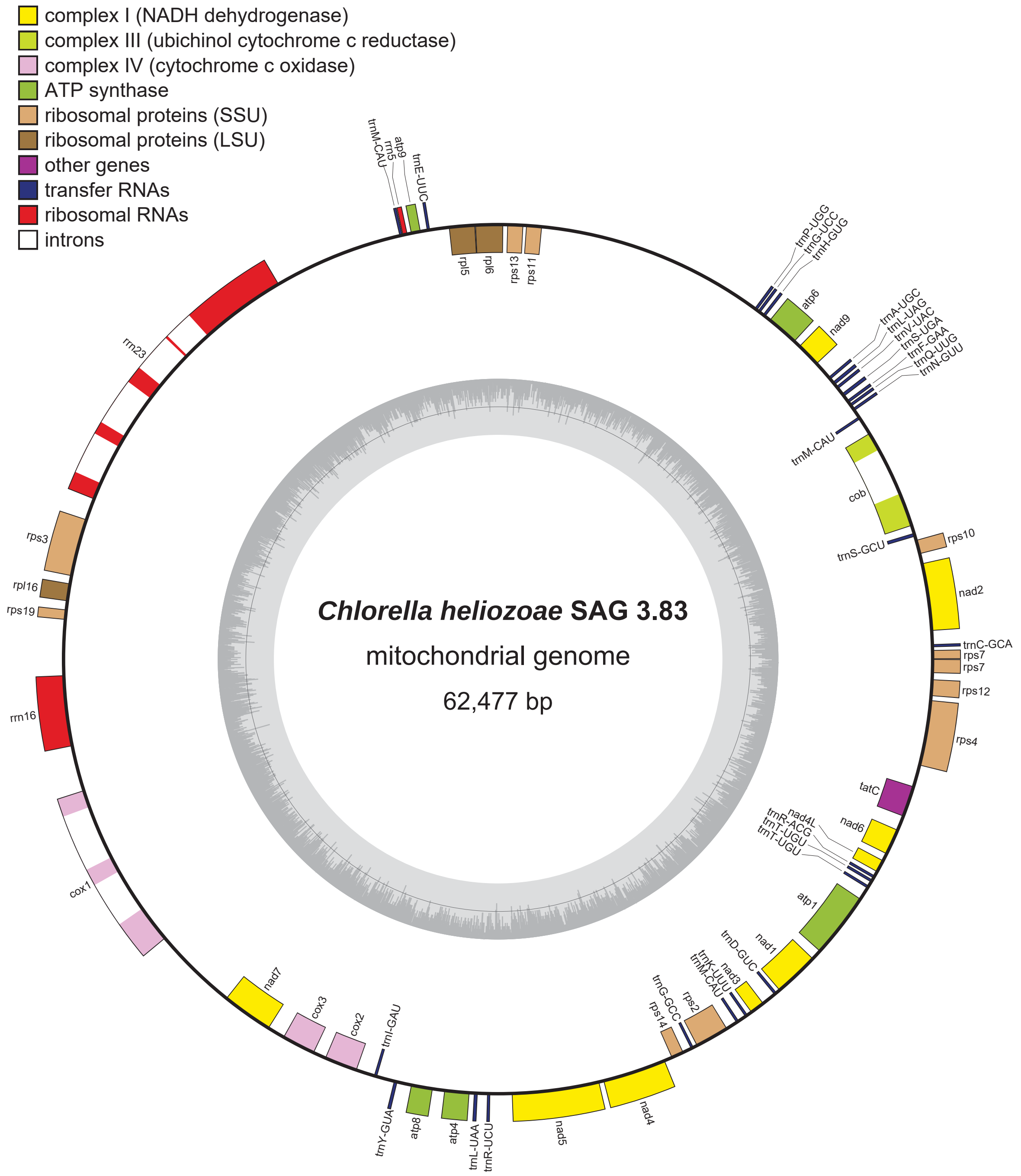

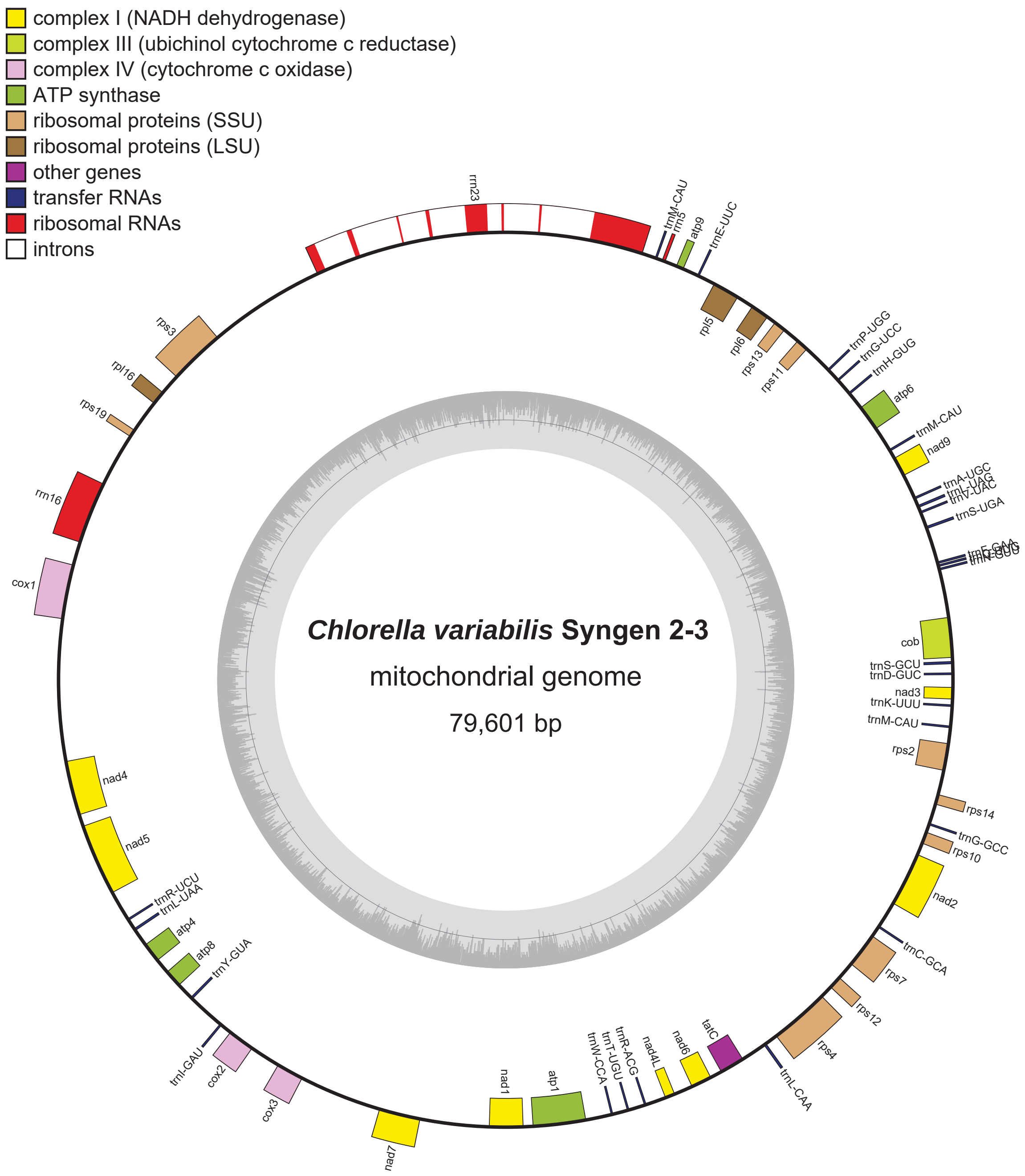

complex I (NADH dehydrogenase)
complex III (ubichinol cytochrome c reductase)
complex IV (cytochrome c oxidase)
ATP synthase
ribosomal proteins (SSU)
ribosomal proteins (LSU)
other genes
transfer RNAs
ribosomal RNAs
introns
Chlorella variabilis Syngen 2-3
mitochondrial genome
79,601 bp
rrn23
trnM-CAU
rrn5
atp9
trnE-UUC
rpl5
rpl6
rps13
rps11
trnP-UGG
trnG-UCC
trnH-GUG
atp6
trnM-CAU
nad9
trnA-UGC
trnL-UAG
trnV-UAC
trnS-UGA
cob
trnS-GCU
trnD-GUC
nad3
trnK-UUU
trnM-CAU
rps2
rps14
trnG-GCC
rps10
nad2
trnC-GCA
rps7
rps12
rps4
trnL-CAA
tatC
nad6
nad4L
trnR-ACG
trnT-UGU
trnW-CCA
atp1
nad1
nad7
cox3
cox2
trnI-GAU
trnY-GUA
atp8
atp4
trnL-UAA
trnR-UCU
nad5
nad4
cox1
rrn16
rps19
rpl16
rps3

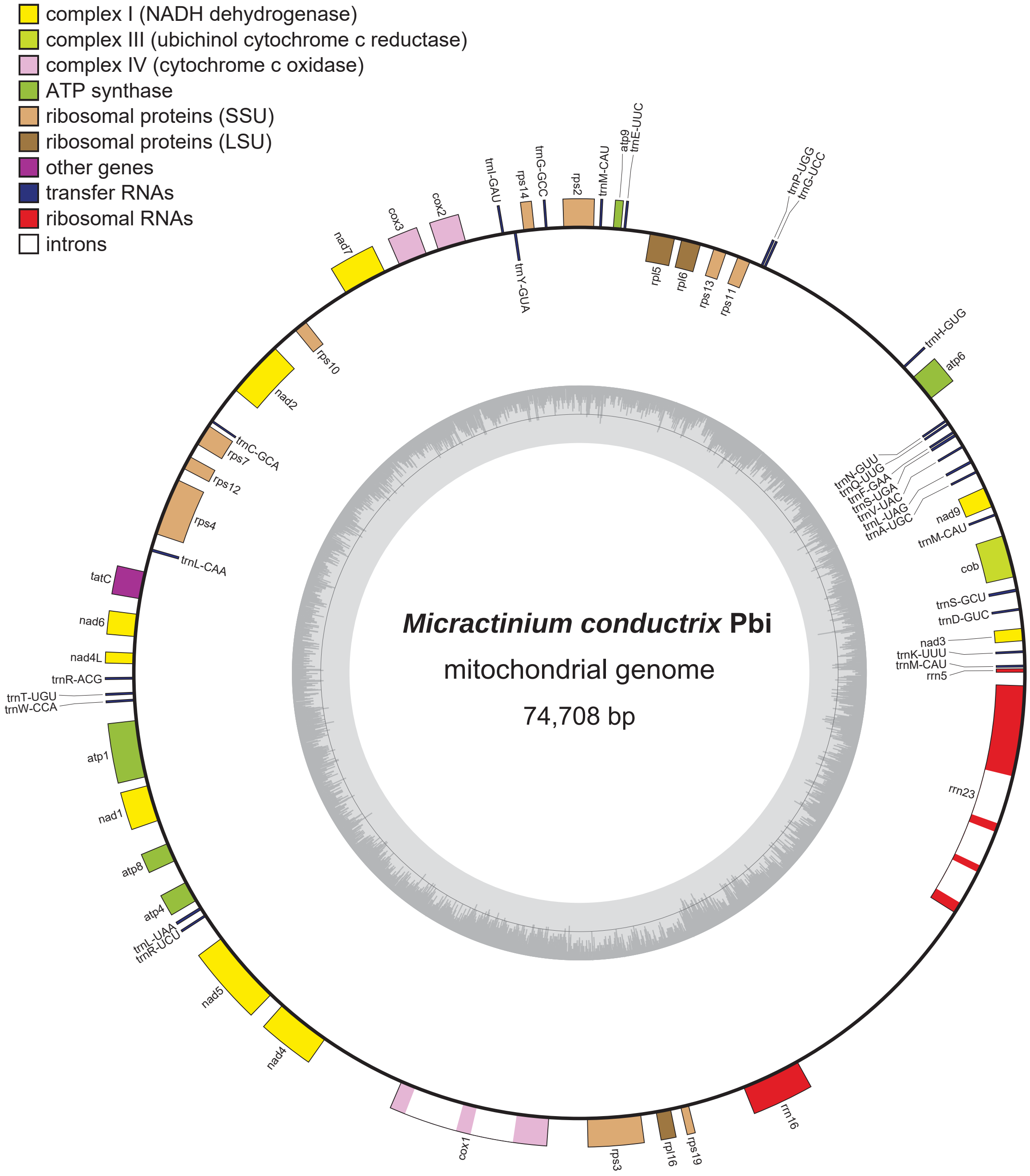
complex I (NADH dehydrogenase)
complex III (ubichinol cytochrome c reductase)
complex IV (cytochrome c oxidase)
ATP synthase
ribosomal proteins (SSU)
ribosomal proteins (LSU)
other genes
transfer RNAs
ribosomal RNAs
introns
Micractinium conductrix Pbi
mitochondrial genome
74,708 bp
nad7
cox3
cox2
trnI-GAU
rps14
trnG-GCC
rps2
trnM-CAU
atp9
trnE-UUC
trnY-GUA
rpl5
rpl6
rps13
rps11
trnP-UGG
trnG-UCC
trnH-GUG
atp6
rps10
nad2
trnC-GCA
rps7
rps12
rps4
trnL-CAA
trnN-GUU
trnQ-UUG
trnF-GAA
trnS-UGA
trnV-UAC
trnL-UAG
trnA-UGC
nad9
trnM-CAU
cob
trnS-GCU
trnD-GUC
nad3
trnK-UUU
trnM-CAU
rrn5
tatC
nad6
nad4L
trnR-ACG
trnT-UGU
trnW-CCA
atp1
nad1
atp8
atp4
trnL-UAA
trnR-UCU
rrn23
nad5
nad4
rrn16
cox1
rps3
rpl16
rps19

Figure S2. Depth of coverage plots for the *C. heliozoae*, *C. variabilis* Syngen and *M. conductrix* mitogenomes. Coverage is plotted logarithmically with base 2. The mean coverage is shown with a dotted red line.

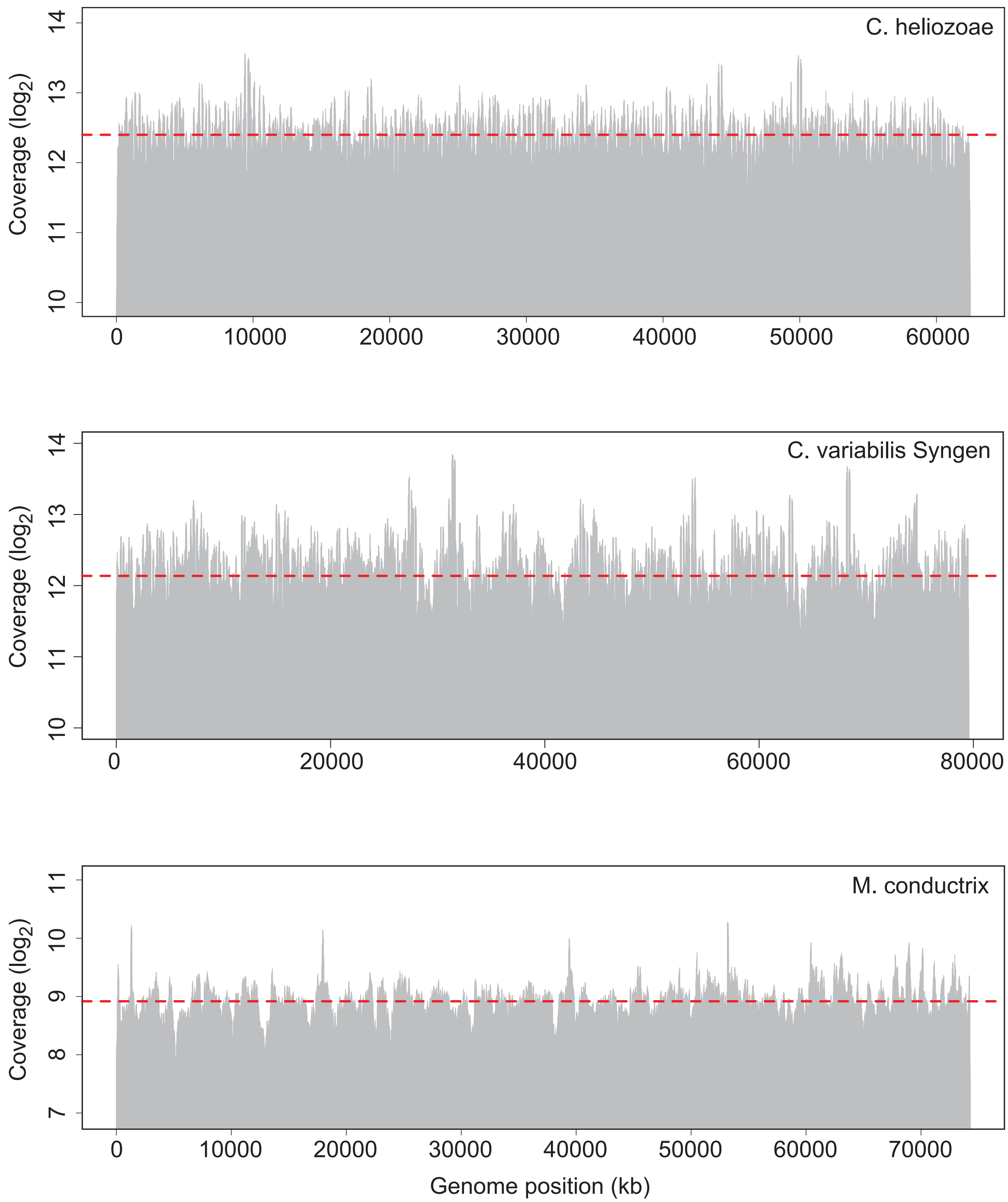

Figure S3A. Sequence alignment for *rrnL* intron 1. Nucleotides in each column are shaded in black when shared by at least 50% of the taxa. Cp: chloroplast intron; Mt: mitochondrial intron. GenBank accession numbers: *C. variabilis* Syngen (KY629618), *Halimeda discoidea* (KX808496), *Tydemania expeditionis* (LN810505).

```
                           10        20        30        40        50        60        70        80        90       100
                   ....|....|....|....|....|....|....|....|....|....|....|....|....|....|....|....|....|....|....|....|
Cvar Syngen_Mt     AGAAAATCCGTTGGAGTTGTAAAATTTGGCTATATGCTGGAAACCCTCGTCTAAATTTAGACTACATAATACTACTTTAATTTTTATGTAACAATGTCGA
Halimeda_Cp        AATCAA-CCGTTGGAGTTATAAAATTTGGCTATATGCTGGAAAATCTCATTG--CTTCTTTTTACA-AAAATAATTTT----------CAGCGAAGCTGA
Tydemania_Cp       AAAGAA-CCGTTGGAGTTATAAAATTTGGCTATATGCTGGAAACTCTCGTTGGAACTTTTTCTACA-AAAATTA------------------------AA

                          110       120       130       140       150       160       170       180       190       200
                   ....|....|....|....|....|....|....|....|....|....|....|....|....|....|....|....|....|....|....|....|
Cvar Syngen_Mt     CTTTTTTTTTAATTTTTAGAATTAA--AATGCGAGACAATCAGCAGGAAAGACTTGATTATTATGAATTTAGTTTTAAATAAAATTTCATTAAATAAAGC
Halimeda_Cp        ACTGTTTTTTG----TCACAATAAAATAAGAAGAGATAATCAGCAGGAAAGACTTTA----TAGAAAAAAATGTTAAAAAATATTCCCGAAAAACATGG
Tydemania_Cp       AGATTTTTTTG----TGACAATGAAA-AAGTAGAGACAATCAGCCGGAAAGATTTTA----AATAAAAATGATTCTTAAAAATATTCCCGAAAAACATGG

                          210       220       230       240       250       260       270       280       290       300
                   ....|....|....|....|....|....|....|....|....|....|....|....|....|....|....|....|....|....|....|....|
Cvar Syngen_Mt     ATATTATATTACTGGATTCGCTGATGGCGAAGGGAGCTTTAATACTAGTTTTCGAAAACGTGATGATTTTTTAATTGGATGGAAAATTACTCCTGTATTT
Halimeda_Cp        CTATTATATTACAGGTTTTGCAGATGGTGAAGGTTCCTTCAATGTTTCTTTTCGGCGTCGAAACGATTATTTGATTGGATGGAAAATAACACCTGTTTTT
Tydemania_Cp       CTACTATATTACCGGTTTTGCTGATGGTGAAGGTTCATTTAACGTATCTTTTCGTAAAAGAAATAATTTTTTAATTAGATGGAAAATAACACCAGTTTTT

                          310       320       330       340       350       360       370       380       390       400
                   ....|....|....|....|....|....|....|....|....|....|....|....|....|....|....|....|....|....|....|....|
Cvar Syngen_Mt     AATATTTCTCAAAAAGAAAGAGATATATTAGCTCTAATCAAAAACCATTTAGAGTGTGGGACTTTACGCTTTAGAAAAGATGGTGTATGGGCTTATGAAG
Halimeda_Cp        AATATTTCACAAGATGAACGTGAAATTTTGGCTTGGATTAAACATATTTTAAAATGTGGAACAATTCGTTTTCGGAAAGATGGTGTTTGGGTTTTTGAAG
Tydemania_Cp       AATATTAGCCAAGATGAACGTGAAATTTTGGCATGGATTAAACACATTTTAAAATGTGG-----------------AGATAAAGTTTGGGTTTTTGAAG

                          410       420       430       440       450       460       470       480       490       500
                   ....|....|....|....|....|....|....|....|....|....|....|....|....|....|....|....|....|....|....|....|
Cvar Syngen_Mt     TTGAAACTCGTAGTGCACTTCATGAAAACATTCTACCTTTTTTTGAAAGATTTCAATTTCTTTCAACTAAAAAGAAAAAAGATTTTAGTAGATTTAAAAA
Halimeda_Cp        TTGATAATCAAAACTCTTTACATAAACAAATTTTGCCTTTTTTTGATCGGTTTCAATTTTTATCAAAAAAAAAAAAATTACAGTATCAAAAATTTAAGAA
Tydemania_Cp       TTACAAATCAACAAGCTTTAAATGAACAAATTCTTCCATTTTTTGATCGATTTCCTTTTCAATCAAAGAAAAAAAA-------TTTCATAAATTTAAAAA

                          510       520       530       540       550       560       570       580       590       600
                   ....|....|....|....|....|....|....|....|....|....|....|....|....|....|....|....|....|....|....|....|
Cvar Syngen_Mt     AATTATAAATGTTTTAGAAGGGAGTAATACTACTACCCTCAATGATTTAAAAGAAATCTTAAAATTAGTTGATGAA-------ATCGAATCTAAGGC---
Halimeda_Cp        AATTTGTAACTTGTTAAATCAAACATCCTCTAATACTTATGAAACTGTTCGACAAATTTTAATTTTAAGAAATGA--------GTCAAATATTAAAATGT
Tydemania_Cp       GATTTGTAA------------------ACCAAATACTTATGAGACGATTCAACAAATTCTTATTTGGAGAAATGAAATAATGTATCCAATCCAAAAAT--

                          610       620       630       640       650       660       670       680       690       700
                   ....|....|....|....|....|....|....|....|....|....|....|....|....|....|....|....|....|....|....|....|
Cvar Syngen_Mt     ----AAGTAGAAAATTTACTAATAATGAAATTTTAGAACGTGCCAATGAATTTTGGTCTAGAAATCAAGAAAAAATTTCTATTTTAAATGCTAAATAA--
Halimeda_Cp        CCAAAAAACGAAAATATACAGATGCTTGTATTTTGCAGCGTGCTGAATTTTATTGGAATAAAAATAAAAAAA-------TCATTCAAATGAAAAA-----
Tydemania_Cp       -TCAACATCGACGATATAGTGATGCTTTAATTTTGCAGCGTGCTAAATTCTATTGGAAGAAAAATCAAGAAA-------TCATTCAGATAAAAAATTATA

                          710       720       730       740       750       760       770       780       790       800
                   ....|....|....|....|....|....|....|....|....|....|....|....|....|....|....|....|....|....|....|....|
Cvar Syngen_Mt     --TCAATTATCCTCAGAGACTAA-ACGCCAAACAATGTCTTTTTAAAAATT--ACATTGATGATAGAGTCCGATCTCTTTAGTGATAAAGAGGGTTTATT
Halimeda_Cp        --TAAAGAATCCTCAGAGACTAATACGCCAAACA------CTTGACATATTCAAGAGTGATGATAGAGTCCGATCTTTCTAGTGATAGAAAGGCTTT--T
Tydemania_Cp       TTTAAAAAATCCTCAGAGACTAATACGCCAAAGA------TCTCA-----TTGAGATT-ATGATAGAGTCCGATCTTTCTCGCGATAGAAAGGAATGTAT

                          810       820       830       840       850       860       870       880       890       900
                   ....|....|....|....|....|....|....|....|....|....|....|....|....|....|....|....|....|....|....|....|
Cvar Syngen_Mt     TGTGGAAAAATTAGTGCTACATGTTTTCTATATCAAATAACCGATTATAATTCAAACAGAATCTTCTGTTTGTACTAATAATTTAACATAGTAGGTAATG
Halimeda_Cp        CGCAAAGCGTCTAA---------------AAATACGATAA--GCTAAAGCTTCA-------------GTTT-------------------TTAGTTAACA
Tydemania_Cp       ATTGAAAAATTAAA---------------AAATAATACATTCGCTAATATTTAA-------------TTTTTTAAAATTTATTAATCTATTAGTTAACA

                   ....|
Cvar Syngen_Mt     -----
Halimeda_Cp        TTAAG
Tydemania_Cp       TTCAG
```

Figure S3B. Sequence alignment for *rrnL* intron 2. Nucleotides in each column are shaded in black when shared by at least 50% of the taxa. Cp: chloroplast intron; Mt: mitochondrial intron. GenBank accession numbers: *C. variabilis* NC64A (KM252919), *C. variabilis* Syngen (KY629618), *C. heliozoae* (KY629615), *C. vulgaris* (AY008337), *Koliella corcontica* (KM462874), *Pedinomonas tuberculata* (KM462867), *Chlorosarcina brevispinosa* (KM462875), *Nephroselmis olivacea* (AF110138).

```
                            10        20        30        40        50        60        70        80        90       100
                    ....|....|....|....|....|....|....|....|....|....|....|....|....|....|....|....|....|....|....|....|
Cvar_NC64A_Mt       TAAATGCATTTCATTTACGCTCGACTTCTTTGAAAATTGGATAAATTTTCAAAGAAGTTAGAATCGCTAGCCTTAGTAAAACTAAACTATATGCTGGAAA
Cvar_Syngen_Mt      TAAATGCATTTCATTTACGCTCGACTTCTTTGAAAATTTATCCAATTTTCAAACAAGTTAGAATCGCTAGCCTTAGTAAAACTAAACTATATGCTGGAAA
Cheliozoae_Mt       TAAATATGATGTATTGACGCTC------CTTGAAGGTACCACC----TTCAAGAC-----AAATCGCTAGCCTTAGTAAAACTAAACTATATGCTGGAAA
Cvulgaris_Mt        TAAATGAGAATCATTTACGCTCA-----CTTGATAGTCGCCTA-----TCAAGAA---CAAACTCGCTAGCCTTAGTAAAACTAAACCATATGCTGGAAA
Koliella_Cp         TGAT------------------------TTTGATAAT----------CACAAATC-------ATCGCTAGCCTTAGTAAAACCAAACTATATGCTGGAAA
Pedinomonas_Cp      TAA----------------------------GATGATATCTTG----CTCAA----------TTCGCTAGCCTTAGTAAAATCAAACTATATGCTGGAAA
Chlorosarc_Cp       TAAA-----------GACGCTCAACTGTTTT----------------TACAGACA------ATTCGCTAGCCTTAGTAAAACTAAACTATATGCTGGAAA
Nephroselm_Mt       TAAC------------------------AATCATAATATGTTA-----TCAGGAA-----TATATGCTAGCCTTATTAAAACCAAACTATATGCTGGAAA

                           110       120       130       140       150       160       170       180       190       200
                    ....|....|....|....|....|....|....|....|....|....|....|....|....|....|....|....|....|....|....|....|
Cvar_NC64A_Mt       CTCCTCAAAAACTGTGTACTACTCGTTAAGATACTT--TCACTCCTTTTTCATTCCAAATTTGAAAAATTTGGAAAGAACCAAGTGAGTGGTTCT--TCA
Cvar_Syngen_Mt      CTCCTCAAAAACTGTGTACTACTCGTTAAGATACTT--TCACTCCTTTTTCATTCCAAATTTGAAAAATTTGGAAACAACCAAGTGAGTGGTTCT--TCA
Cheliozoae_Mt       CTCCTCAAAAACTTTGTACTACTCGTCAAGA-----------------------------------------------------------ATTCT--TC-
Cvulgaris_Mt        CTTCTCAAAAACTTTCTACTACTCGTAA--------------------------------------------------------------GTGTG--TCA
Koliella_Cp         CTCCTCAAAAACTTTTAGTACTCGTT----------------------------------------------------------------TTTTT--TTA
Pedinomonas_Cp      CTCCTCCAAAACTTCTTTGTACTCATCCCAA-----------------------------------------------------------ATTTT--TTT
Chlorosarc_Cp       CTCCTCCAAAACTTATCAGTACTAG---------------------------------ATCTACAAAGCATGGTCAGTAAAAATCTGAAAGTTTTACTCA
Nephroselm_Mt       CTCCTCCAGAAATCTTTTCATTTTGACCAAATGATTCGCAATTGATTTCTTTGACTTAAGTCAAACAGTCTTGTCAT-------------ACTCG--TTA

                           210       220       230       240       250       260       270       280       290       300
                    ....|....|....|....|....|....|....|....|....|....|....|....|....|....|....|....|....|....|....|....|
Cvar_NC64A_Mt       ATACATGTATTGAAGAACAAATCAAGTAAATTTTTGAAAGCTCTTTATGAAAATCAAAGGCGTTTTCTAAAACACTTTTCAGTAAAAATGTACAA-----
Cvar_Syngen_Mt      ATACATGTATTGAAGAACAAATCAAGTAAATTTTTGAAAGCTCTTTATGAAAATCAAAGGCGTTTTCTAAAACACTTTTCAGTAAAAATGTACAA-----
Cheliozoae_Mt       ---------------------------------------------------------------------------TTTTCAGTAAAAATGTACAA-----
Cvulgaris_Mt        ACAC------------------------------------------------------------------------TTTCAGTGAAAATGTAAAA-----
Koliella_Cp         A----------------------------------------------------------------------------TTCAGTAAAAATCTAAAA-----
Pedinomonas_Cp      G---------------------------------------------------------------------------GCATAGTAAAAATCAAAAA-----
Chlorosarc_Cp       ATTTTAAAATTGAG-----------------------------------------------------TAAAACCTTGGTGACTAAAAAATAGTAACTTTG
Nephroselm_Mt       TTTCCTTAATTTAGTTATTATTTGATTAGGTTACTGAAA-------------------------------------TAGCAGTGAAAATTGACAG-----

                           310       320       330       340       350       360       370       380       390       400
                    ....|....|....|....|....|....|....|....|....|....|....|....|....|....|....|....|....|....|....|....|
Cvar_NC64A_Mt       GGCAAGGGGACAATCAGCAGGAAAGATTTTTGAGGAAATTCTTTCAATCCAAAATCTCCAGATTTTATACTAAATTTTTGAAATTTAGAATAAGAAAGAG
Cvar_Syngen_Mt      GGCAAGGGGACAATCAGCAGGAAAGATTTTTGAGGAAATTCTTTCAATCCAAAATCTCCAGATTTTATACTAAATTTTTGAAATTTAGAATAAGAAAGAG
Cheliozoae_Mt       GACACGGGGACAATCAGCAGGAAAGACTCACACCAAGT-------------GGAGTTCCAGATTTGGAACCAATTTGTTAAAGGAAAGACAAAAAGAATG
Cvulgaris_Mt        GACACGGGGACAATCAGCAGGAAAGATTTTTGG------------------------------------------TGTAAAAATCCAA------------
Koliella_Cp         GACAAGGGGACAATCAGCAGGAAAGACTTATAA--------------------------ATTTTTTATTTTTATTTTTTAT-------------------
Pedinomonas_Cp      GACATGGGGACAATCAGCAGGGAAGACTTTC------------------------------------------TCTTTCAAAGA----------------
Chlorosarc_Cp       GACATGGGGACAATCAGCAGGGAAGACCAATGAAACAA--------------------------------------------------------------
Nephroselm_Mt       AACATGGGGACAATCAGCAGGAAA---------------------------------------------CCAAATAACTCAACTTGAGTTAAAG------

                           410       420       430       440       450       460       470       480       490       500
                    ....|....|....|....|....|....|....|....|....|....|....|....|....|....|....|....|....|....|....|....|
Cvar_NC64A_Mt       -------AATCACTCAAAAAAATCCCCAGAGACT-ATACGTTTGGGGGTTTT------TTGTTGCAAAAAA---AAAGAATT---TATGAATTTACAAAC
Cvar_Syngen_Mt      -------AATCACTCAAAAAAATCCCCAGAGACT-ATACGTTTGGGGGTTTT------TTGTTGCAAAAAA---AAAGAATT---TATGAATTTACAAAC
Cheliozoae_Mt       CTTTTGTCACCACATGTGAGAATCCCCAGAGACT-ATACGTTTGGGGACTTT-------TTTCGCTAAAAA---AAACATC----TATGAATTTACAAAC
Cvulgaris_Mt        -----------------AAAAATCCTCAGAGACT-TTACGTTTGGGGATCTT------TCTGTACTTAAAC---AAACAACTCGATATGAATTTACAAAC
Koliella_Cp         -----------------AAGAATCCTCAGAGACTAATACGTTTGGAGATT----------------AAAAA---TGACAAC----CAT--ACTTA-ATGC
Pedinomonas_Cp      -----------------AAGAACCCTCAGAGACT-ATACGTTTGGAGATATT------------------------TCGATT---TATGAACTTAGAAGC
Chlorosarc_Cp       --------------CATTGGAACCCTCAGAGACT-ATACGTTTGGAGGC-------------------------AAAGAT-----CATGAATCTTGAACC
Nephroselm_Mt       -----------------TAGGATCCTCAGAGACT-ATACGTTTGGGAATTTTCACAGCTTTCAACTAAAAAAGTAAAGAATT---AATGAATTTAAATGC

                           510       520       530       540       550       560       570       580       590       600
                    ....|....|....|....|....|....|....|....|....|....|....|....|....|....|....|....|....|....|....|....|
Cvar_NC64A_Mt       AGAATGGATAGTAGGATTTGTTGATGGTGAAGGATGTTTTCACATTGGAATCAATCAGAACAACACTTTGAAATGTGGATACCAAGTTCTTCCTGAATTT
Cvar_Syngen_Mt      AGAATGGATAGTAGGATTTGTTGATGGTGAAGGATGTTTTCACATTGGAATCAATCAGAACAACACTTTGAAATGTGGATACCAAGTTCTTCCTGAATTT
Cheliozoae_Mt       ACAATGGATTGTAGGTTTTGTTGATGGTGAAGGCTGTTTTCATGTTGGAATCAACAAAAACAGCAGTATCAACACTGGGTATCAAGTTCTGCCTGAATTT
Cvulgaris_Mt        ACAATGGATTGTAGGATTTGTTGATGGAGAAGGATGTTTCCATGTAGCAATCAACTCTCAAAAAACATCCAAGCTCGCCTTTCAAGTGCTCCCAGAATTT
Koliella_Cp         TGAGTGGATAGTAGGTTTTGTTGATGGAGAAGGATGTTTCCATATCGACATTAATAAAAATAACCTTATGAAACTTGGTTATCAAGCACTTCCAGAATTT
Pedinomonas_Cp      ACAATGGATTGTTGGATTTGTGGATGGAGAAGGTTGTTTTCATATAGCAATCAATTCGAATCAAGAAATGAAACTTGGAGTTCAAGTTCTTGCAGAATTC
Chlorosarc_Cp       ACAATGGATTGTTGGTTTTGTTGATGGTGAAGGATGCTTTTTTGTTGGATTCAACAAACAATCTACAATGAAAATAAAAATACAAGTTTTACCCGAGTTT
Nephroselm_Mt       TCAATGGATTGTAGGTTTTGTAGATGGTGAGGGTTGTTTTCATATCGAAATGAATCCTCAACCATCGATGAAAATGAAATGTCAAATATTATGTAGTTTT

                           610       620       630       640       650       660       670       680       690       700
                    ....|....|....|....|....|....|....|....|....|....|....|....|....|....|....|....|....|....|....|....|
Cvar_NC64A_Mt       ACAGTCACACAACATAAACGAGATATTCAAGTACTTCATGCACTTAAAAAATATTTTGGATGTGGTGTTGTACGAGTGAATCATGGTGATCGATATTGTT
Cvar_Syngen_Mt      ACAGTCACACAACATAAACGAGATATTCAAGTACTTCATGCACTTAAAAAATATTTTGGATGTGGTGTTGTACGAGTGAATCATGGTGATCGATATTGTT
Cheliozoae_Mt       ACAGTGACACAACACGAAAGAAATCTCAAAGTGCTGTACGCTTTGAAATCCTATTTTCAGTGTGGTGTGGTCCGTGTGAATCATGGCGATCGATTTTGTT
Cvulgaris_Mt        ACTATAACCCAACATAAACGTGACGTTCAAGTGTTGTATGGGTTAAAAAATTTTTTTGGTTGCGGCGTGGTGCGTGTCAATCATGGTGATCGTTATTGTT
Koliella_Cp         ACCGTTGTTCAACACGAATCCGATGTACAAATTCTTAATGCCTTGAAAGCCTTTTGGAAATGTGGTGTGGTTCGTAAAAATCACGGAAATAGAATGTGTT
Pedinomonas_Cp      GTAGTAGTTCAGCACGAAAGTGATCGGCAAATTTTAGAAGCTCTCAAAGCCTTTTGGGGTTGCGGAGTGGTTCGCAAAAATAACAAGGATTGTCTTTGTT
Chlorosarc_Cp       ACAGTAGTTCAACATCAACGAGATATTGCAGTACTGCAAAACTTAAAAACTTATTTTCAATGTGGGGTTGTTCAAAGAAACCATAGTGATCGATACGCAT
Nephroselm_Mt       GTCATAACTCAACATATACGTGATATTCAATTATTGCATGCGATTAAAGACTATTTTGGTTGTGGAGTTGTTCGTCGCGATAAAGAAAATATATATTGTT
```

```
                            710       720       730       740       750       760       770       780       790       800
                    ....|....|....|....|....|....|....|....|....|....|....|....|....|....|....|....|....|....|....|....|
Cvar_NC64A_Mt       ATCGGGTTCGTGGATTTCAACATCTTCAAAAAATTATAGTTCCTTTTTTTGAAAAACACAAGTTACTTACTAAGAAACGTGTTGATTTTGAAAAATTTCG
Cvar_Syngen_Mt      ATCGGGTTCGTGGATTTCAACATCTTCAAAAAATTATAGTTCCTTTTTTTGAAAAACACAAGTTACTTACTAAGAAACGTGTTGATTTTGAAAAATTTCG
Cheliozoae_Mt       ATCGTGTTCGAAATTTTGACCATTTACACCACATCATTCTTCCTTTTTTTGAGACACATAAATTAGTGACCTCAAAACGCATCGATTTTGAAAAATTTCG
Cvulgaris_Mt        ATCGTGTTCGCGGGTTTCCTCATCTTCGAGACATCATTCTGCCATTTTTCGAAAAACACAAGTTACTTACTAAGAAACGTGTTGATTTTGAAAAATTTAG
Koliella_Cp         ATCGAGTACGTAATTTAAACCATCTTCAAACAATAATAATTCCTTTTTTTGAAAAACATAAACTTAAAACAAAAAAACGTATCTCTTTTGAACGTTTTCG
Pedinomonas_Cp      ATCGCGTGCGCGATCTAGAACAATTAAAAACAAGAATTGTTCCTTTTTTTGAAAAACATAAATTAAAAACAAAAAAACGCATTGATTTTGAAAAATTTCG
Chlorosarc_Cp       ATCGTGTACGAGGTCATGAAAATCTCCTTAAAAAAATTATACCTTTTTTTGAAAAACATAAACTCAAAACGAAGAAACGAGTTGATTTTGAAAAGTTTAG
Nephroselm_Mt       ATCGTGTTCGATCTTTTCAACATTTACGAACTATTATAATACCATTCTTTGAGAAACATGAATTAAAAACGAAGAAACATGTAGATTTTCTGAAGTTTCG

                            810       820       830       840       850       860       870       880       890       900
                    ....|....|....|....|....|....|....|....|....|....|....|....|....|....|....|....|....|....|....|....|
Cvar_NC64A_Mt       TACTGTAATAAATATGATAGAAGAAAAACGTCACCTCACTTCAGAAGGATTAGTAAAAATTCGTGAAATTCAACAAACCATGAATACTAAAGAGAACAAA
Cvar_Syngen_Mt      TACTGTAATAAATATGATAGAAGAAAAACGTCACCTCACTTCAGAAGGATTAGTAAAAATTCGTGAAATTCAACAAACCATGAATACTAAAGAGAACAAA
Cheliozoae_Mt       ACGAATTTGTCTGTTAATGGAACGAAAAGCACACCTTTCTGTCCAAGGAGTAGAAGAAATTCGCCAAATTCAAGAGTCTATGAATCGAAAAGGAA-----
Cvulgaris_Mt        AAAGGTGATTCTGAAAATGAATTGCGGAGATCATTTAACTCCTCAAGGAATCGATGAAATTCGCAAAATTCAAGAAACCATGAATAGAAAAGGAATCAAG
Koliella_Cp         GTCGGCAGTTTTATTAATGGAAAAAAAAGAACATTTAACTTTAGAAGGTTTAGATAAGCTACGTAAATTAAGAGAAGGTATAAATAG-------------
Pedinomonas_Cp      TGATGTAATTGCTCTTATGGATCAACAAGCTCACTTAACTCTAGAAGGAGTCGAAAAAATTAATCAAATTCGAAAGACTATGAATCGCAAAG--------
Chlorosarc_Cp       AGATATTGTTCTCTTAATGGAAAAAAAAGCTGATCTTCAATTTGAGGGTTTAGAAAAAATCACCAAAATCGCAAATACCATGAATACAAAAGGGG-----
Nephroselm_Mt       AAAAGTGATTTTAATGATGGAGAAAAAACTTCATCTAGAAAAAGAAGGTTTTGAAAGAATTCAACAAAT------AGTTCTAGCCGGAAGAAAG------

                            910       920       930       940       950       960       970       980       990      1000
                    ....|....|....|....|....|....|....|....|....|....|....|....|....|....|....|....|....|....|....|....|
Cvar_NC64A_Mt       TCTCAAAATTTTTAAATTTGA--TAATTTC--TTTTTTTTTTTTTTTAAGAACTAAATAAAACAAACTCAACCAATTCTTAAAGCACA---AAAAATCTA
Cvar_Syngen_Mt      TCTCAAAATTTTTAAATTTGA--TAATTTC--TTTTTTTTTTTTTTTAAGAACTAAATAAAACAAACTCAACCAATTCTTAAAGCACA---AAAAATCTA
Cheliozoae_Mt       --------------------------------------TCCGTTTTTAAGATCAAGAGCAA------------------------------AAAAGTCTA
Cvulgaris_Mt        TTC-----TTTGAAAAATCAAGTTGATCTCAAAATTATTCTAATTTTGAGAAGAAAACACGAAATGGAC------------AAGTACATTGATAGATCTA
Koliella_Cp         --------------------------------------TCTTTCT-------------------------------------------------AATCTA
Pedinomonas_Cp      -------------------------------------------------------------------------------------------GAATATCTA
Chlorosarc_Cp       ----------------------------------TTTCAGTCACTTCAAATACAAAATGGAGAGAAGAACAGATTCACTCGAA--------TAAAGCCTA
Nephroselm_Mt       -----------TCAAATTCGAAGGAGTTTCACGAAAATTCGGGATTAGATAATAAGGTTGACGTAAGTG------------AAGAAAGGAAGTAGATTTA

                           1010      1020      1030      1040
                    ....|....|....|....|....|....|....|....|..
Cvar_NC64A_Mt       AGATAGAGTCCAGCTTTTTATGAAAATAAAAAGATT-AACTG
Cvar_Syngen_Mt      AGATAGAGTCCAGCTTTTTATGAAAATAAAAAGATT-AACTG
Cheliozoae_Mt       AGATAGAGTCCAGCTTTTAATGAAAATTAAGAGAGT-ATCTG
Cvulgaris_Mt        AGATAAAGTCCAGCTCTTTATGAAAATAAAGAGATT-AACTG
Koliella_Cp         AGATAGAGTCCACCTTACCATGAAAATGGATAGATT-TAGTG
Pedinomonas_Cp      AGATAGAGTCCAGCTCATCATGAAAATGATAAGATTATACTG
Chlorosarc_Cp       AGATAGAGTCCAGCTTTGGTTGAAAAATCAGAGATAGAACTG
Nephroselm_Mt       AGATAGAGTCCAGCTTTGATTGAAAGATCAGAGAGA-AACTG
```

Figure S3C. Sequence alignment for *rrnL* introns 5 and 7. Nucleotides in each column are shaded in black when shared by at least 50% of the taxa. Cp: chloroplast intron; Mt: mitochondrial intron; i5: intron 5 homolog; i7: intron 7 homolog. GenBank accession numbers: *C. variabilis* NC64A (KM252919), *C. variabilis* Syngen (KY629618), *Micractinium conductrix* (KY629619), *Auxenochlorella protothecoides* (KC843974), *Lobosphaera incisa* (KP902678), *Prototheca wickerhamii* (U02970), *Gloeotilopsis sarcinoidea* (KX306821), *Pedinomonas tuberculata* (KM462867), *C. heliozoae* (KY629615), *Dicloster acuatus* (KM462885).

```
                            10        20        30        40        50        60        70        80        90       100
                    ....|....|....|....|....|....|....|....|....|....|....|....|....|....|....|....|....|....|....|....|
Cvar_NC64A_Mt-i5    TAA-----------------------------TTCTGTTAGTCCTCGTTTTTGTGAGAAGAACGTGCGTTTCTTGAGAAAACCTCTCAAGATAAAAAG
Cvar_Syngen_Mt-i5   TAA-----------------------------TTCTGTTAGTCCTCGTTTTTGTGAGAAGAACGTGCGTTTCTTGAGAAAACCTCTCAAGATAAAAAG
Micractinium_Mt-i5  TAA-----------------------------ATCTGTTAGTCCTAGTTCTTCGTGAGAAGAAAA-GCGTTTCTTGAGGCTGTACTCAAGATAAAAAGAA
Auxenochlor_Mt-i5   TCAA----------------------------AGTTTGTAGTCCCGCTTTGTAGCGATACAAG------------------------------GGCAATA
Lobosphaera_Mt-i5   TAAATAAGCAAGAAAGGTTTCATTTGAAACCTACCTGTTAGTCCCATTTCTTCGCGAGAAGAA--------------------------AAAAAAAAAAA
Prototheca_Mt-i5    TTA------------------------------GTTTGTAGTCCCGCTTTATAGAGATATAAG------------------------------GGCAATA
Gloeotilopsis_Cp    GAA---------------------------------AGTTGTCCCGCCAGGCGGTGACGCCAG---------------------------GATAAAAACA
Pedinomonas_Cp      AAAA--------------------------------TCTTGTCCCCTATTTTTGTGAAAAAAT-----------------------------ATGGAAAA
Cvar_NC64A_Mt-i7    TACAA---------------------------AAGCCATAGTCGAGGCT-------------------------------------------------AA
Cvar_Syngen_Mt-i7   TACAA---------------------------AAGCCATAGTCGAGGCT-------------------------------------------------AA
Cheliozoae_Mt-i7    TAACAA--------------------------AAGCCATAGTCGAGGCT-------------------------------------------------AA
Prototheca_Mt-i7    TAATTTTAGC----------------------AGAAGATAGTCGAGGCT-------------------------------------------------AA
Dicloster_Cp        TGGCATTT------------------------------TAGTCGAGGCT-------------------------------------------------AA

                           110       120       130       140       150       160       170       180       190       200
                    ....|....|....|....|....|....|....|....|....|....|....|....|....|....|....|....|....|....|....|....|
Cvar_NC64A_Mt-i5    ACTGGGTGAATTGCAAGAACGCTTCA-----------------------------------------------------------GTGATGATCATCACC
Cvar_Syngen_Mt-i5   ACTGGGTGAATTGCAAGAACGCTTCA-----------------------------------------------------------GTGATGATCATCACC
Micractinium_Mt-i5  ACTGGGTGAATTGCAAGAACGCTAAA--------------------------------------------------GTGTTTTTAGAGACTGAAAACATC
Auxenochlor_Mt-i5   ACTGGGTGAATTGCAAGAAGGCTAAG------------------------------------------GTAAAATAAAACCGATTGGTTTAATATTTACT
Lobosphaera_Mt-i5   ACCGGGTGAATTGCAAGAATATCTAAAGAGAAAAATTACAACCCCCTTTTTTTTCTTTTTTTTGTATAAAAAAAAAGAAAGCGGGGGAAAAATTTGTTTG
Prototheca_Mt-i5    ACTGGGTGAATTGCAAGAAGGCTGTG-----------------------------------------------TAAACATTATATAATAATATTTGTTTA
Gloeotilopsis_Cp    ACTGGGTGAATTGCAAAAATAGCTAA-----------------------------------------------------------GTGATGATAATTCAT
Pedinomonas_Cp      ACTGGGTGAATTGCAAGAAAGCTAAG-----------------------------------------------------------GGTATTTTTATACTT
Cvar_NC64A_Mt-i7    AGTGGGTGAATTGCAAGAATCTCTTT----------------------------------------------------------------------TTCG
Cvar_Syngen_Mt-i7   AGTGGGTGAATTGCAAGAATCTCTTT----------------------------------------------------------------------TTCG
Cheliozoae_Mt-i7    AGTGGGTGAATTGCAAGGACCTCTGT---------------------------------------------------------------------TTCTG
Prototheca_Mt-i7    AGTGGGTGAATTGCAAGGATCTCTCT------------------------------------------------------TTAATTTAGAATTTAACTTG
Dicloster_Cp        AGTGGGTGAATTGCAAGGAACTCTCG---------------------------------------------------------------------ATTTC

                           210       220       230       240       250       260       270       280       290       300
                    ....|....|....|....|....|....|....|....|....|....|....|....|....|....|....|....|....|....|....|....|
Cvar_NC64A_Mt-i5    AT-----------------------------------------------------------------GCCAACTTGCAGCGAAGCATTTCAAAAGGATAC
Cvar_Syngen_Mt-i5   AT-----------------------------------------------------------------GCCAACTTGCAGCGAAGCATTTCAAAAGGATAC
Micractinium_Mt-i5  AT-----------------------------------------------------------------GCCAACTTGCAGCGAAGCATTTTGAATGGAAAA
Auxenochlor_Mt-i5   AT-----------------------------------------------------------------GCTAATTTGCAGCGAAGTTCTTCGAATAGGCTC
Lobosphaera_Mt-i5   ATAAACAGAAAAAAACAGAGAACAAATCACTGATTTGTTCCTGTTAGGAAGAGTAGTTTTTCGTCCAGACAATTTGCAGCGAAGTCTCTTGAAAGGTATA
Prototheca_Mt-i5    TT-----------------------------------------------------------------GCTAATTTGCAGCGAAGTTCTTCGAATAGATAA
Gloeotilopsis_Cp    AT-----------------------------------------------------------------GCCAATTTGCAGCGAAGCTCTTTGAAAACACAA
Pedinomonas_Cp      AT-----------------------------------------------------------------GCTAACTTGCAGCGAAGCTTATTGAACATGCAA
Cvar_NC64A_Mt-i7    ATTACCCGAGAAAAA----------------------------------------------------GACAATTTGCAGCGAAGCATTGTGAATGGAAAA
Cvar_Syngen_Mt-i7   ATTACCCGAGAAAAA----------------------------------------------------GACAATTTGCAGCGAAGCATTGTGAATGGAAAA
Cheliozoae_Mt-i7    GAAACCCGAGAAACA----------------------------------------------------GACAACTTGCAGCGAAGCATTGTGAATGGAAAA
Prototheca_Mt-i7    ATTAAGA------------------------------------------------------------GACAATTTGCAGCGAAGCATTTCGAATAGAATT
Dicloster_Cp        CTGAACCAGAAACGA----------------------------------------------------GACAATTTGCAGCGAAGCTTCTCGAAAGGAAAC

                           310       320       330       340       350       360       370       380       390       400
                    ....|....|....|....|....|....|....|....|....|....|....|....|....|....|....|....|....|....|....|....|
Cvar_NC64A_Mt-i5    TAC----------------------------------------------------------TGTAGAAATGAACGTTCAACGACTAGAGAGTGAGAATTT
Cvar_Syngen_Mt-i5   TAC----------------------------------------------------------TGTAGAAATGAACGTTCAACGACTAGAGAGTGAGAATTT
Micractinium_Mt-i5  ACA----------------------------------------------------------AGTAAAAATGAACGTTCAACGACTAGAGAATGAGGACTT
Auxenochlor_Mt-i5   TAAAAAAA-----------------------------------------------------TGTAGAAGAAAACGTTCAACGACTAGAGAATGAGGATTT
Lobosphaera_Mt-i5   AAACAGATTTTTTCTGTATATAAAATT----------------------------------GGTAAAGAGGAACGTTCAACGACTAGGAAATGAGGATAA
Prototheca_Mt-i5    ATTAATAAATAATAAACTTTGTTAAAAACAAAAA-------AAAACGCAGTTTATTTATATTGTAGAAGAAAACGTTCAACGACTAGAGAATGAGGATAT
Gloeotilopsis_Cp    ATTAATTAATAATACTA--------------------------------------------AGTAAAAGAGAACGTTCAACGACTAGAAAGTGAGAGTGT
Pedinomonas_Cp      TTAAAAAAA----------------------------------------------------TGTAAATAAGAACGTTCAACGACTAGAGAGTGAGGATTT
Cvar_NC64A_Mt-i7    AAAA---------------------------------------------------------AGTAACAATGAACGTTCAACGACTAGAGAGTGAGAACTT
Cvar_Syngen_Mt-i7   AAAA---------------------------------------------------------AGTAACAATGAACGTTCAACGACTAGAGAGTGAGAACTT
Cheliozoae_Mt-i7    AAAA---------------------------------------------------------AGTAACAATGAACGTTCAACGACTAGAGAGTGAGAATTT
Prototheca_Mt-i7    TTTGCAT------------------------------------------------------AGTAGAAATGAACGTTCAACGACTAGAGAATGAGGATAT
Dicloster_Cp        AAATTCCCTTTCCTTTCCTTTCCAAATTAACGAAGTTAATGAAAGAAAAGAGAAAGAGAGCAGTAGAGATGAACGTTCAACGACTAGAGAATGAGGATTT

                           410       420       430       440       450       460       470       480       490       500
                    ....|....|....|....|....|....|....|....|....|....|....|....|....|....|....|....|....|....|....|....|
Cvar_NC64A_Mt-i5    CC--TATCAATAATCTCTCCACGAGCGCCCAGCAAC---TCTCTTTACAGACTTT---------------------------------------------
Cvar_Syngen_Mt-i5   CC--TATCAATAATCTCTCCACGAGCGCCCAGCAAC---TCTCTTTACAGACTTT---------------------------------------------
Micractinium_Mt-i5  CC--TACCAATAAATTCTCCACGAGCGCCCAGTAAC---TCAAAAAAATAACATTCT---------------------------------------TTTA
Auxenochlor_Mt-i5   CT--TACCAATAATTTCTCCACGAGCGCCCAGCAAC---TT-----------------------------------------------------------
Lobosphaera_Mt-i5   CC--TACCAATAAATTTCCCACGAGCGCCCGGCAAC---TTTATTATTTAGCCCACCCTAAAAAAAAAGAGGGGGCTAAAAATGTTCCACCACTTCGTTA
Prototheca_Mt-i5    CT--TACCGATAATTTCTCCACGAGCGCCCGGCAAC---TT-----------------------------------------------------------
Gloeotilopsis_Cp    GT--AACCAATAATCTTTCCACGAGTGCCCAGCAAC---TTAATTAATT---------------------------------------------------
Pedinomonas_Cp      GCAAAACCAATAAACTCTCCACGAGTGCCCGGCTTC---TT-----------------------------------------------------------
Cvar_NC64A_Mt-i7    CC--TATCAATACCCTCTCCACGAGCGCCCGCTACC---ATATTTTTTGAATTATGAGTAAA--------------------------------------
Cvar_Syngen_Mt-i7   CC--TATCAATACCCTCTCCACGAGCGCCCGCTACC---ATATTTTTTGAATTATGAGTAAA--------------------------------------
Cheliozoae_Mt-i7    CC--TATCAATAATCTCTCCACGAGCGCCCGCTACCATTTTTTTTTTTGAAAGTATGACCAAA-------------------------------------
Prototheca_Mt-i7    CT--TACCAATAATTTCTCCACGAGCGCCCGCTAAC---TT-----------------------------------------------------------
Dicloster_Cp        CC--TACCAATAATTTCTCCACGAGCGCCCACTAAC---TTTTCATTCA-----------------------------------------------TTTA
```

```
                              510       520       530       540       550       560       570       580       590       600
                     ....|....|....|....|....|....|....|....|....|....|....|....|....|....|....|....|....|....|....|....|
Cvar_NC64A_Mt-i5     -----------ATGACATAAAA--------ACCAACACAATTTCAGAGAACCCTTCTTGAAGAATATAAAAAA-------------------------AA
Cvar_Syngen_Mt-i5    -----------ATGACATAAAA--------ACCAACACAATTTCAGAGAACCCTTCTTGAAGAATATAAAAAA-------------------------AA
Micractinium_Mt-i5   TGAAAAAACCAGTAGCCACACA--------AAGAAAACGGTTAAAGTGAATCTTAATTACTTATAATTAAGAA--------------AAGCAACAAATTT
Auxenochlor_Mt-i5    ----------------AAAAAA--------ACAAACATGTTATATAAATCATTACGTGGTAAAGTATTAAAAA---------------------------
Lobosphaera_Mt-i5    AAAAACTATGCTTAACAAAATG--------AATGATACCCCCTTTAAACCCCTAAGTGGTAAGAAATCAAAAATTTGTTCCACCAAAACAAAAAGCAGTT
Prototheca_Mt-i5     ---------------------------------------------------------------ATATTAAAAT---------------------------
Gloeotilopsis_Cp     -----------TTAATTAAAAC------------------------------------GCTAAATTTGAAAAG---------------------------
Pedinomonas_Cp       ------------------------------------------------------------------TCAAAAA---------------------------
Cvar_NC64A_Mt-i7     -----------CTTATAAAAAACACAAGAGATGGATATCATTTGAATTCTCCCTATTGGCAAAATTATAAAAA-------------------------AA
Cvar_Syngen_Mt-i7    -----------CTTATAAAAAACACAAGAGATGGATATCATTTGAATTCTCCCTATTGGCAAAATTATAAAAA-------------------------AA
Cheliozoae_Mt-i7     -----------ATCATAAAAAACACAAGAGATGGCTATCATTTGAATTCATCCTATTGACAAACTTATAAAAA-------------------------GA
Prototheca_Mt-i7     -----------------------------------------------------------------GTAAAGAA---------------------------
Dicloster_Cp         CAAGACTAACTATGATAAAAAATAGACGGCATGGATACCATTTAAATTCTTCTTATTGGCAAAACTACAAAAA-------------------------AA

                              610       620       630       640       650       660       670       680       690       700
                     ....|....|....|....|....|....|....|....|....|....|....|....|....|....|....|....|....|....|....|....|
Cvar_NC64A_Mt-i5     CCTCTCTTTAT---------------CACAACATCTTTTTGATATAAGTATAGGGACGCTTCTAGGTGATGCCTCAATTCAAACTCAAAATGGTGGAAAA
Cvar_Syngen_Mt-i5    CCTCTCTTTAT---------------CACAACATCTTTTTGATATAAGTATAGGGACGCTTCTAGGTGATGCCTCAATTCAAACTCAAAATGGTGGAAAA
Micractinium_Mt-i5   CTTTTCTTTTTTTATTAAAAAAAGAACACAAACGCTTTTTGAAATTGCTGTTGGTGGCCTTTTAGGTGACGTTTCAATTCAAACTCAAAATCAAGGCAAA
Auxenochlor_Mt-i5    ----------------------------------------------------------------------------------------------------
Lobosphaera_Mt-i5    CCTTCTCTCCCCTTT-----------CACAAGAGGTGTTTGAAGTAGCAATAGGTACAGTTTTAGGTGATGCTAGTGTACAAACTCAAAACAATGGTAAA
Prototheca_Mt-i5     ----------------------------------------------------------------------------------------------------
Gloeotilopsis_Cp     ----------------------------------------------------------------------------------------------------
Pedinomonas_Cp       ----------------------------------------------------------------------------------------------------
Cvar_NC64A_Mt-i7     CTCTCCCAAAATTAC-----------CGCAAGACTGTTTTGATACAGCCGCAGGAATGATTTTAGGTGATGCCACAATGTAGTACGCTAGTCGTGAAGCA
Cvar_Syngen_Mt-i7    CTCTCCCAAAATTAC-----------CGCAAGACTGTTTTGATACAGCCGCAGGAATGATTTTAGGTGATGCCACAATGTAGTACGCTAGTCGTGAAGCA
Cheliozoae_Mt-i7     CTCTTCCAAAACTCC-----------CACAAGACTGTTTTGATACAGCTGTAGGAATGCTTTTGGGTGATGCTACAATGTCTTATGTCAGTCATAACGCA
Prototheca_Mt-i7     -------------------------------------CTCTAAATAAATGAAATGATAAATTTAG-----------------------------------
Dicloster_Cp         CAGTGCCTGATTTGT-----------CAGACACTCAATTTCAAATTGCCGTTGGTATGATTTGTGAAGATGCTACCATGAGGAGAGTGAGTAAAGAGGCG

                              710       720       730       740       750       760       770       780       790       800
                     ....|....|....|....|....|....|....|....|....|....|....|....|....|....|....|....|....|....|....|....|
Cvar_NC64A_Mt-i5     ACATATCGATTGAAATTTCAACAATCGGATGCTCGTCACCGTGACTATTTATGGCATTTACATAGTCTCTATTCTGAGTGGGTTCTTTCTTCACGACCTT
Cvar_Syngen_Mt-i5    ACATATCGATTGAAATTTCAACAATCGGATGCTCGTCACCGTGACTATTTATGGCATTTACATAGTCTCTATTCTGAGTGGGTTCTTTCTTCACGACCTT
Micractinium_Mt-i5   CCTTATCGACTTCAATTTCAACCAGCGGATTATTTACACCGTGACTATGTATTTCATTTACATCAAAAATGGAGTGATTGGGTTCTTTCTC---CTCCTT
Auxenochlor_Mt-i5    ----------------------------------------------------------------------------------------------------
Lobosphaera_Mt-i5    ACTTATCGTTTAAAGTTTTCACAAA---GTAGCAAACATAAAGATTATTTGTTTGATTTACATGAAATTTTTCGTGATTGGGTATTATCACCCCCACATT
Prototheca_Mt-i5     ----------------------------------------------------------------------------------------------------
Gloeotilopsis_Cp     ----------------------------------------------------------------------------------------------------
Pedinomonas_Cp       ----------------------------------------------------------------------------------------------------
Cvar_NC64A_Mt-i7     TTGATAAAATTTGAGCAAGGGGTTAAACAAAAAGAATTTCTGTTTCATCTTTTCGATTTGTTTTCAAGGTATTGTTTTATGGTAGAGCCTG---GAATCA
Cvar_Syngen_Mt-i7    TTGATAAAATTTGAGCAAGGGGTTAAACAAAAAGAATTTCTGTTTCATCTTTTCGATTTGTTTTCAAGGTATTGTTTTATGGTAGAGCCTG---GAATCA
Cheliozoae_Mt-i7     TTGATTAAATTTGAACAAGGGGTCAAACAAAAAGAATTTCTGTTTCATCTTTTTGATGTGTTTTCTAGGTATTGTTTTATGACAGAACCTGGAAGACGAG
Prototheca_Mt-i7     ----------------------------------------------------------------------------------------------------
Dicloster_Cp         TTTATTAAGTTTGAGCAAGGTTACAAACAGAAAGACTTTGTTTTTCATCTTTTTGATAATTTTGGAGGCTATACTTTTGTGGATTCCCCAAAAACTCGTT

                              810       820       830       840       850       860       870       880       890       900
                     ....|....|....|....|....|....|....|....|....|....|....|....|....|....|....|....|....|....|....|....|
Cvar_NC64A_Mt-i5     TTTATGACCCAACACAAGCTATGTGGTCATTTC------AAACGATTTCTCATAGTGAATTTTGCTCATTAGCAACACTTTTTATTCTTGATCGTGACGG
Cvar_Syngen_Mt-i5    TTTATGACCCAACACAAGCTATGTGGTCATTTC------AAACGATTTCTCATAGTGAATTTTGCTCATTAGCAACACTTTTTATTCTTGATCGTGACGG
Micractinium_Mt-i5   TTTTCGATAATGATCGTAAAATGTAGAGTTTCC------AAACAATTTCACATGTTAATTTTGTCAAATTGGCTGAGCTTTTCGTTCTAGATGAGAATGG
Auxenochlor_Mt-i5    ----------------------------------------------------------------------------------------------------
Lobosphaera_Mt-i5    TTAATGA---AAAACGTAATATGTGGAGTTTTC------AAACCAAGGCAGATCAAGAATTTAAAAAAATAGCAACCCTCTTTTTTGATCTTGATCCTAA
Prototheca_Mt-i5     ----------------------------------------------------------------------------------------------------
Gloeotilopsis_Cp     -------------------------------------------------------CGAATTTA-------------------------------------
Pedinomonas_Cp       ----------------------------------------------------------------------------------------------------
Cvar_NC64A_Mt-i7     GATATGAAAAAGATAGAAATGTGAAAAGTTATTGGTTTAAAACGTTTTCTTTTCCTGATTTTACTCATTTATTT---CTTTTAATTCACGAAAAAATCAA
Cvar_Syngen_Mt-i7    GATATGAAAAAGATAGAAATGTGAAAAGTTATTGGTTTAAAACGTTTTCTTTTCCTGATTTTACTCATTTATTT---CTTTTAATTCACGAAAAAATCAA
Cheliozoae_Mt-i7     T---TGAAAAAAATCACTGTGTGAAAAGTTATTGGTTTAAAACCTTTTCATTTCCTGACTTTACTGATTTATTT---CTGATACTTCACGACCAACGTCA
Prototheca_Mt-i7     ----------------------------------------------------------------------------------------------------
Dicloster_Cp         TTGATGATAAGAAAAAAAAAATCAAAAGTTACTGGTTTAGAACTTTCTCACATAAAAATTTTACACCACTCTTTGAATCATTTTATAAACTTCGACTTGA

                              910       920       930       940       950       960       970       980       990      1000
                     ....|....|....|....|....|....|....|....|....|....|....|....|....|....|....|....|....|....|....|....|
Cvar_NC64A_Mt-i5     AAATCGAAGCCAAAAACATATTCAACACGGCCTTATTGAGAAATATCTCACACCTCTTGAAAATTGGACCAATTTTCAAAAAGGTCTTGCGTATTGGTTT
Cvar_Syngen_Mt-i5    AAATCGAAGCCAAAAACATATTCAACACGGCCTTATTGAGAAATATCTCACACCTCTTGAAAATTGGACCAATTTTCAAAAAGGTCTTGCGTATTGGTTT
Micractinium_Mt-i5   TGTGAAATGTAAAAAACATCTCAAACCTTCTTTAATTTAAAAAGAC----------------------------------CAGGTTTAGCCTATTGGTTT
Auxenochlor_Mt-i5    ------------------------------------------------------------------------------------------ATTATAAATT
Lobosphaera_Mt-i5    TGGGAGTAAGAAAAAACAAATAATGCCTTG---TATTGAAAAATATATCACACCTC------------------------GTGTACTAGCCTATTGGTTT
Prototheca_Mt-i5     ----------------------------------------------------------------------------------------------------
Gloeotilopsis_Cp     --------------------------------------------------------------------------------------TTAACTATCATATT
Pedinomonas_Cp       ----------------------------------------------------------------------------------------------------
Cvar_NC64A_Mt-i7     TGGAAAATGGAAAAAAAGAATAAAGGAAGGACTTGTTCGTGACTTTTTAACTCCAA------------------------AAGGATTAGCTTATTGGATT
Cvar_Syngen_Mt-i7    TGGAAAATGGAAAAAAAGAATAAAGGAAGGACTTGTTCGTGACTTTTTAACTCCAA------------------------AAGGATTAGCTTATTGGATT
Cheliozoae_Mt-i7     TGGAAAATGGAAAAAAACAATCAAGCAAGGATTGATTCGTGATTGGTTAACTCCTC------------------------GAGGATTCGCGTATTGGATT
Prototheca_Mt-i7     ---------------------------------------------------------------------------------------------TTATTAT
Dicloster_Cp         TGGAAAGTATCGGAAAACAATCACTCCAAATCTCGTTTTTGACCTAGTCACTCCAA------------------------GAAGCTTAGCTTATTGGATT
```

```
                          1010      1020      1030      1040      1050      1060      1070      1080      1090      1100
                    ....|....|....|....|....|....|....|....|....|....|....|....|....|....|....|....|....|....|....|....|
Cvar_NC64A_Mt-i5    ATGGACGATGGAGGAAGATTAAGTTATAATAAGGACTATGAACGAAAAGGATTTGTCTTAAACACTCATAGTTTTTCTCAAAATCAAGTAGAACTGTTGT
Cvar_Syngen_Mt-i5   ATGGACGATGGAGGAAGATTAAGTTATAATAAGGACTATGAACGAAAAGGATTTGTCTTAAACACTCATAGTTTTTCTCAAAATCAAGTAGAACTGTTGT
Micractinium_Mt-i5  ATGGATGATGGTGAAAAATCGAGTGATACTAGAGATTCTCCTCGAAGAGTCTTTGCACTTAATACACAAGGTTTTACAGAAAATGAAGTTGAAATGTTGT
Auxenochlor_Mt-i5   ATGGATGATGGAAGTAAATTATGTTATAATAAGGATTATCCTCGTAAAGGGTTTGTCCTTAATACGCAAGGATTTAAAACAGCTGATGTCATTTTAGCAT
Lobosphaera_Mt-i5   ATGGATGATGGTGGAAAAGCATGTTATAACAAAGATTTTCCGAGAAAAGGTTTAGTTTTTAATACTCAGGGGTTTTCTGAGGATCATGTAATTTTTTTAG
Prototheca_Mt-i5    ----------------------------------------------------------------------------------------------------
Gloeotilopsis_Cp    ATGGCATATA------------------------------------------------TTCCTCCTTCAATTCCTCCTTCGCATCGATTAGTT-------
Pedinomonas_Cp      ----------------------------------------------------------------------------------------------------
Cvar_NC64A_Mt-i7    ATGTGTGATGGAAGTCTGCAAAAAGATAACCTT---------------ACGCTGATTCTTGATACTCAAAGTTTTTCTTTACAGGAAAATTCTGTTTTAT
Cvar_Syngen_Mt-i7   ATGTGTGATGGAAGTCTGCAAAAAGATAACCTT---------------ACGCTGATTCTTGATACTCAAAGTTTTTCTTTACAGGAAAATTCTGTTTTAT
Cheliozoae_Mt-i7    ATGTGTGATGGAAGTTTACAAAAAGACAACCAA---------------ACACTGATTCTTCATACTCAAGGATTTTGTTTGCAGGAGAACACTGTTTTAT
Prototheca_Mt-i7    AACTATAATAAATGAAATTTATTTTAGAACAGCAAAAAAAATAGCAAAAAAATTCTTAGAAATACTAA------------AGATGGATTCAATTCTTT--
Dicloster_Cp        ATGTCTGATGGGTCATTACAAAACGACAGCAAG---------------TCAATGATTTTACATACGCAGTCCTTCACAGAAAAGGAAAATTATATAGTCA

                          1110      1120      1130      1140      1150      1160      1170      1180      1190      1200
                    ....|....|....|....|....|....|....|....|....|....|....|....|....|....|....|....|....|....|....|....|
Cvar_NC64A_Mt-i5    GTCAAGGTTTAGAGGAAAAATTTCGATTAAAG------TGTTGGCCTAAACAAAACAAAAAAAATGG------ATCATTGTGATCTCAGGTCATGATCAT
Cvar_Syngen_Mt-i5   GTCAAGGTTTAGAGGAAAAATTTCGATTAAAG------TGTTGGCCTAAACAAAACAAAAAAAATGG------ATCATTGTGATCTCAGGTCATGATCAT
Micractinium_Mt-i5  GTGATAGATTAACCAAAAAATTTGGGTTGAAA------TGTTGGAAAAAACCTACTAAA---AATGGTTTT--GTTGTTGTGATTTCTACTGAAAGTTAT
Auxenochlor_Mt-i5   CT-----------CAAGAAATTTTTGTTAAATTTAATATAATTACAAAAATAAGATAAAATAAAAATTTCGCAGTAATTGTTATACCTGCCATTCATCAC
Lobosphaera_Mt-i5   TGAACATTTTAAAAAATAAATATCAGTTAAAT------TGTTGGCATAAACCTAATAAA---AAAGGTTTT--ATTATAGTGATTTCAGGAAATCATTAT
Prototheca_Mt-i5    -------------------ATATTAATTA-----------------------------------------------------------------------
Gloeotilopsis_Cp    -------------------ATTCCACCTAGAA------TAACAAGTCAAACTCGAGGAATTGAACTTTTGA--AAAATTGATTTTT--------------
Pedinomonas_Cp      ----------------------------------------------------------------------------------------------------
Cvar_NC64A_Mt-i7    CAACAGAATTAAATAAGAAATTTTCTCTCCAT------TATCGTGTGATTTTACATAAAACACACTATTAG--GTGATTGAATTTCCTCGTCAAGATAGT
Cvar_Syngen_Mt-i7   CAACAGAATTAAATAAGAAATTTTCTCTCCAT------TATCGTGTGATTTTACATAAAACACACTATTAG--GTGATTGAATTTCCTCGTCAAGATAGT
Cheliozoae_Mt-i7    CAACAGAATTAAACAAGAAATTTCAACTGCAT------TCTCGTGTGATTTCACATAAAACACACTATTTT--GTGCTTGAATTCCCTCGTCACGACAGT
Prototheca_Mt-i7    -------TTCATATAATAAACTTCGTTTA---------TTTATATGGAACAAAAATAA------------------------------------------
Dicloster_Cp        GCGATGAGTTAAACAGAAAGTTTGGCTTTCAT------TCAGTAGTCAAGACCCATAAAACCAACTATTTT--GTGGTTTTTTTTCCGGCTCAAGACGCA

                          1210      1220      1230      1240      1250      1260      1270      1280      1290      1300
                    ....|....|....|....|....|....|....|....|....|....|....|....|....|....|....|....|....|....|....|....|
Cvar_NC64A_Mt-i5    GCGAAGATGTTGGATTTAATCGGAGAGTATGTGATTCCATCTATGCGCCATAAAGTCCCAAAA--------AGCACAGA-----GTTGATGACATAGTCT
Cvar_Syngen_Mt-i5   GCGAAGATGTTGGATTTAATCGGAGAGTATGTGATTCCATCTATGCGCCATAAAGTCCCAAAA--------AGCACAGA-----GTTGATGACATAGTCT
Micractinium_Mt-i5  GAACTTGTGATGAGTTTACTAGATCCATCTAGTATTCCATCAATGCGTCATAAATTACCTTTTGGTACCACATTTTTGA-----GTTGATGACATAGTCT
Auxenochlor_Mt-i5   TATAAATTTGTAAACTACATAAAACCTTATATTGTTCCATCTATGATTTATAAAATAACACTT-----------TAAAA-----GTTGATGACATAGTCT
Lobosphaera_Mt-i5   AATAAATGGAGGGGTTTAGTAGACCCTTATCTCCATCCATCAATGGTTTATAAATTGCCCCTG------ATATAATAAA-----GTTGATGACATAGTCT
Prototheca_Mt-i5    ----------------TAATAAATTAATATTTGTT---------------------------------------AATGA-----GTTGATGACATAGTCT
Gloeotilopsis_Cp    -----------------------------------------------------------------------------AA-----GTTGATGACATAGTCT
Pedinomonas_Cp      ------------------------------------------------------------------------------A-----GAAGATGACATAGTCT
Cvar_NC64A_Mt-i7    GAGAACCTCGCTGCTCTAATCAAACAGTATCTCATCCCAAGTATGCTTTATAAACTTCCCAAT---------TCAAAAAAGTAAGGTAATGACATAGTCT
Cvar_Syngen_Mt-i7   GAGAACCTCGCTGCTCTAATCAAACAGTATCTCATCCCAAGTATGCTTTATAAACTTCCCAAT----TCAAAAAAGTAA-----GGTAATGACATAGTCT
Cheliozoae_Mt-i7    GAGACATTGGGTGCTTTACTGAATCAGTATTTCATCCCAATTATGCTCTATAAACTTCCTGATTGCAACTTGAAAAAAAAATGTGGTAATGACATAGTCT
Prototheca_Mt-i7    --------------TTTATTAAACTAATATGTTCTT-----AATAATTCATAA--TTGCTATTTTAATTAGAGTACAA------GTTAATGACATAGTCT
Dicloster_Cp        CAACTCCTGTTTGATCTTATCCAACCTTTCATGATTCCTTCTATGTGGTATAAATTACCCAAA---------ATGAAAA-----GTTAATGACATAGTCT

                          1310      1320      1330      1340      1350
                    ....|....|....|....|....|....|....|....|....|....|....|....
Cvar_NC64A_Mt-i5    GAACTCTATGGAAACATAGAGAAATAAAGGATAAAGAGCCTTTATGGTAACACAATTG-
Cvar_Syngen_Mt-i5   GAACTCTATGGAAACATAGAGAAATAAAGGATAAAGAGCCTTTATGGTAACACAATTG-
Micractinium_Mt-i5  GAACTTTATGGAAACATCAAGAAATAAAGGATAAAGAGCCTTTATGATAACACGTTTG-
Auxenochlor_Mt-i5   GAACTATATAGAAATATATAGAAGTAAAATATAAACAA-TTTTACGATAACACAATTG-
Lobosphaera_Mt-i5   GAACTTTATGGAAACATAAAGAAATAAAGGATAAAGAGCCTTTATGATAACAAAATTG-
Prototheca_Mt-i5    GAACTAAATGGAAACATTTAGAAGTAAAAAATAAAGAGTTTTTACGGTAACATAATTG-
Gloeotilopsis_Cp    GAGCTTTTAAGAAATTAAAAGATACAAAAGATAAAGAGCTTTTGTGATAACAACA-TG-
Pedinomonas_Cp      GAACTTTTGGGAAATCAAAAGAAACTAGAGATAAAGAGCTCTGGTAGAAACAT-TTTG-
Cvar_NC64A_Mt-i7    GAGCTACTCTGTGAAGAGTAGAAATTTAGGATAAAGAGCCTAAATGATAACATAACTG-
Cvar_Syngen_Mt-i7   GAGCTACTCTGTGAAGAGTAGAAATTTAGGATAAAGAGCCTAAATGATAACATAACTG-
Cheliozoae_Mt-i7    GAGCTACTCTGTGAAGAGTAGAAATTTAGGATAAAAAGCCTAAATGATAACATCACTGG
Prototheca_Mt-i7    GAGCTACTTTGTGAAGAGTAGAAATTTAGGATAAAGAGCCTAAATGATAACATAACTG-
Dicloster_Cp        GAGCTGCTCCGTGAGGAGTAGATATTTAGGATAAAGAGCCTAAATGATAACAAAACTG-
```

Figure S4. Plastome maps of *C. heliozoae*, *C. variabilis* Syngen and *M. conductrix*. Outer genes are transcribed counter-clockwise; inner genes are transcribed clockwise. Gene and intron colors correspond to the functional categories listed in the key at the top left. GC content is shown on the inner circle by dark grey bars. The map was drawn with OgDraw (http://ogdraw.mpimp-golm.mpg.de/).

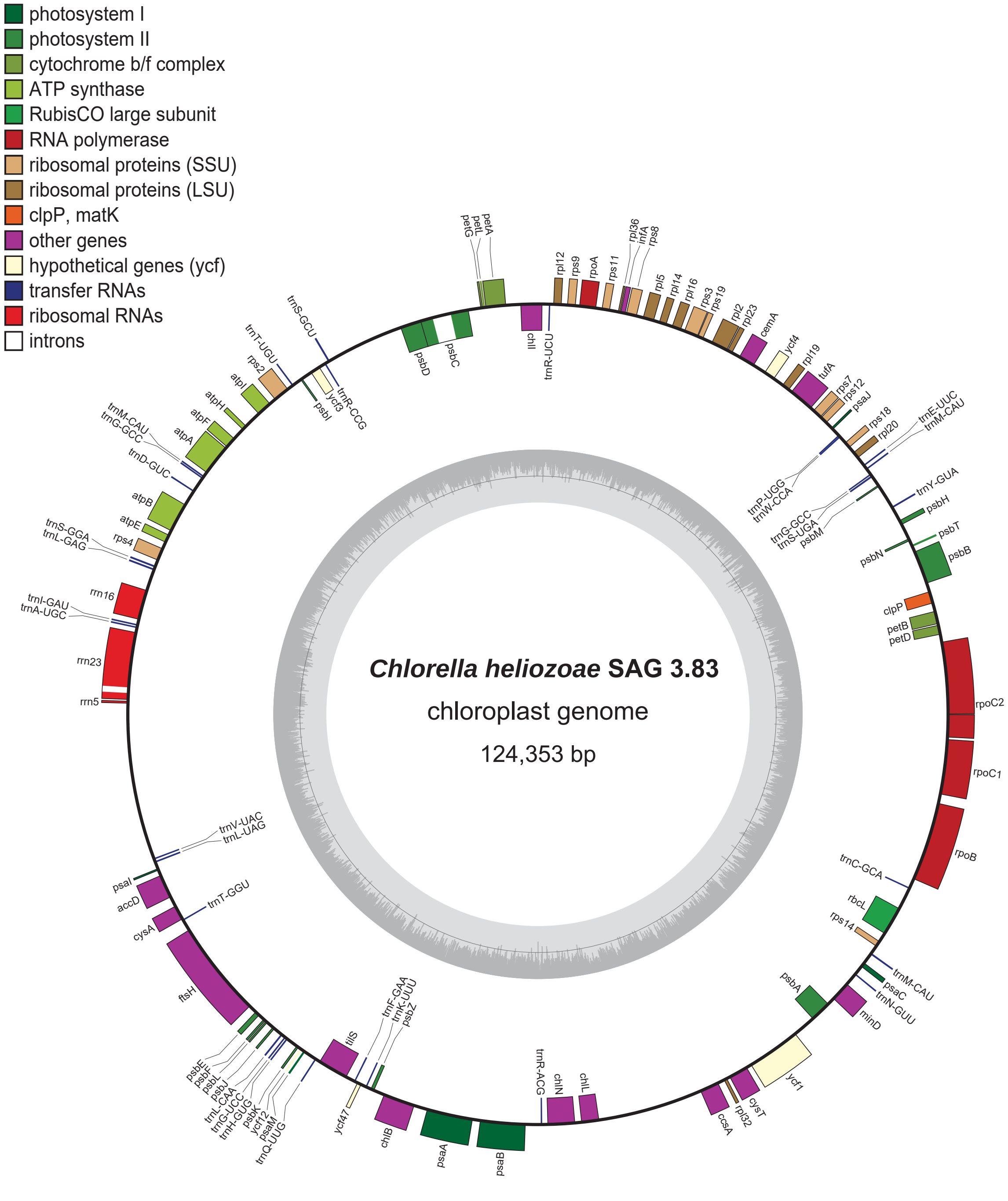

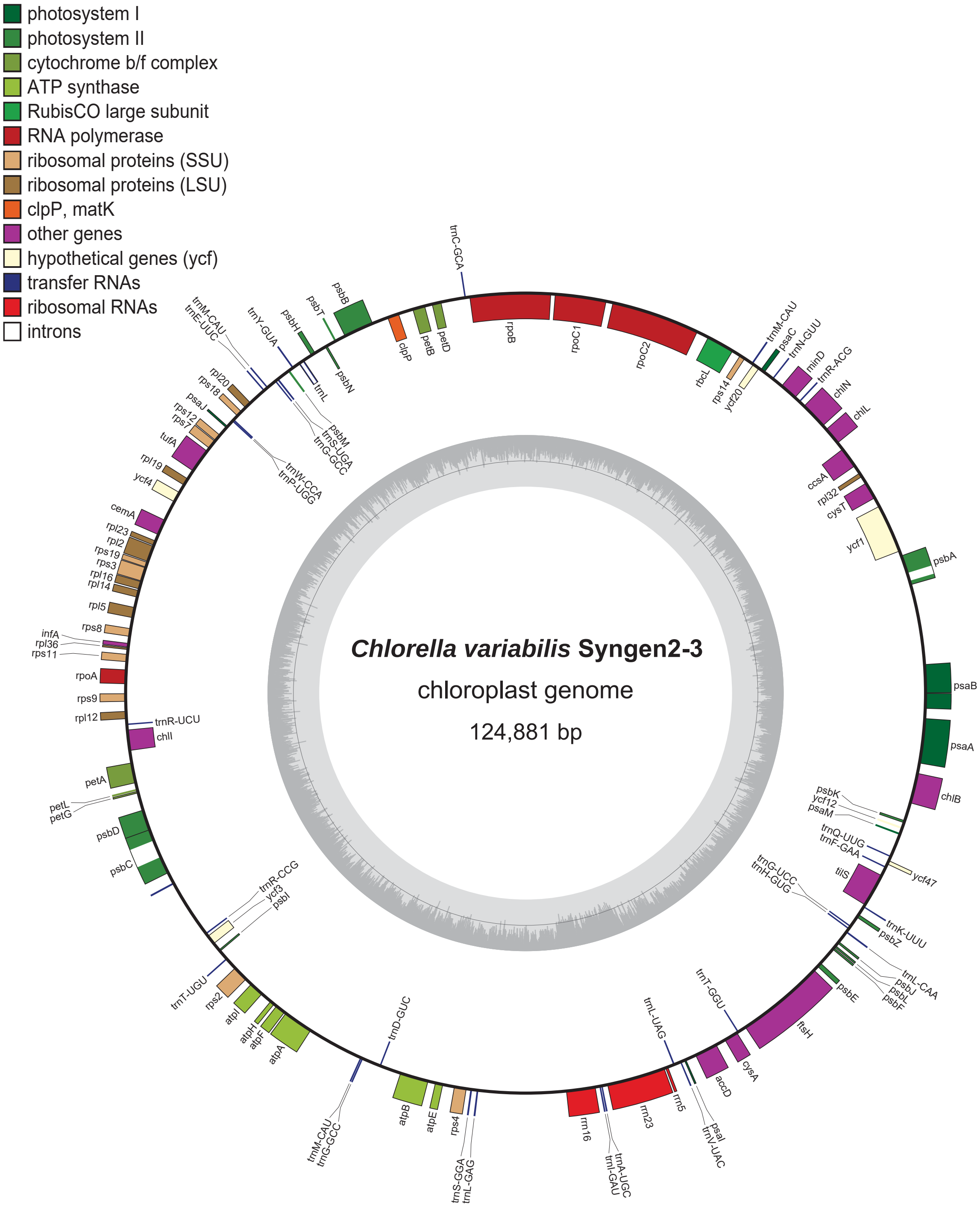

photosystem I
photosystem II
cytochrome b/f complex
ATP synthase
RubisCO large subunit
RNA polymerase
ribosomal proteins (SSU)
ribosomal proteins (LSU)
clpP, matK
other genes
hypothetical genes (ycf)
transfer RNAs
ribosomal RNAs
introns
Chlorella variabilis Syngen2-3
chloroplast genome
124,881 bp

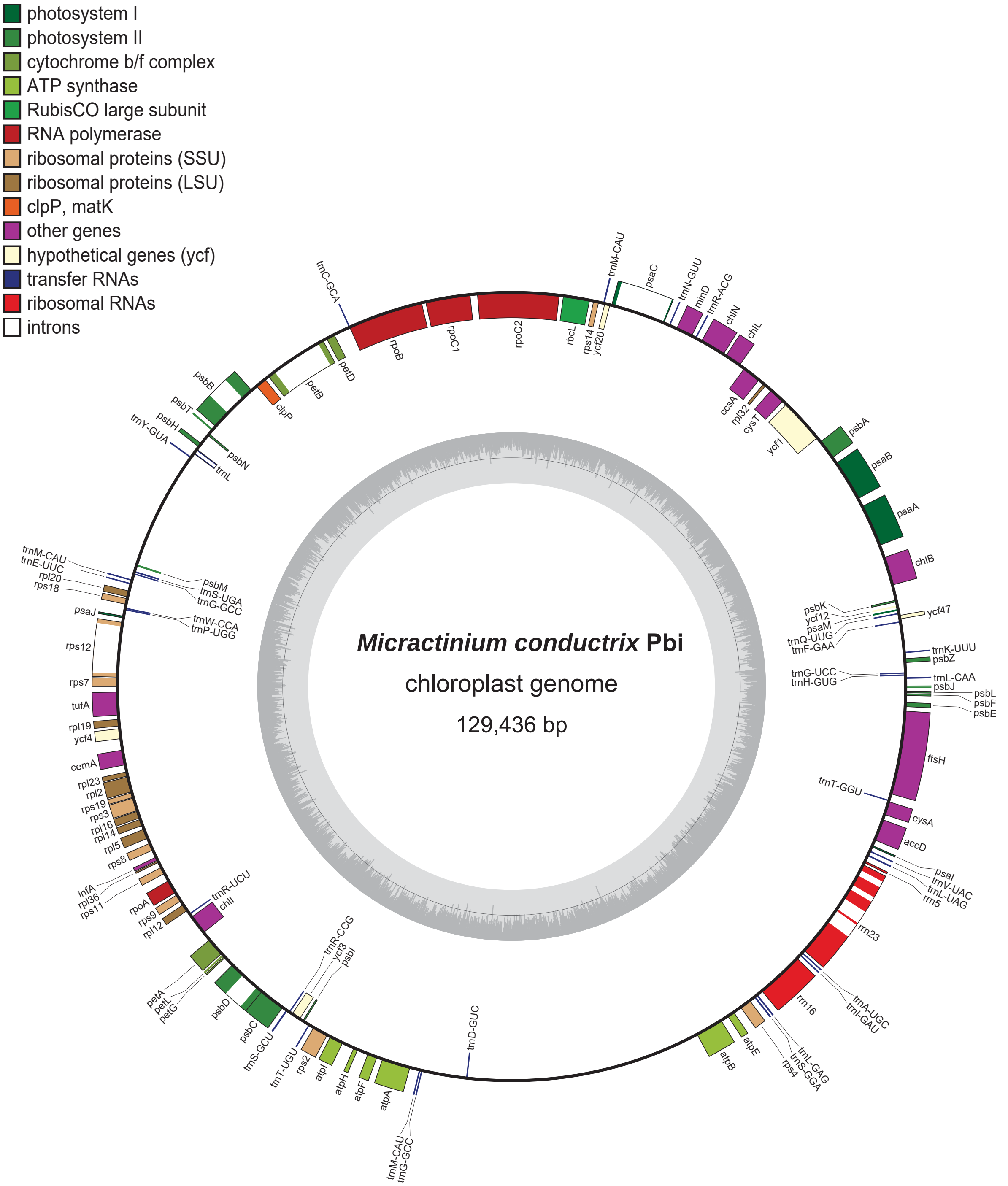
photosystem I
photosystem II
cytochrome b/f complex
ATP synthase
RubisCO large subunit
RNA polymerase
ribosomal proteins (SSU)
ribosomal proteins (LSU)
clpP, matK
other genes
hypothetical genes (ycf)
transfer RNAs
ribosomal RNAs
introns
Micractinium conductrix **Pbi**
chloroplast genome
129,436 bp

Figure S5. Depth of coverage plots for the *C. heliozoae*, *C. variabilis* Syngen and *M. conductrix* plastomes. Coverage is plotted logarithmically with base 2. The mean coverage is shown with a dotted red line.

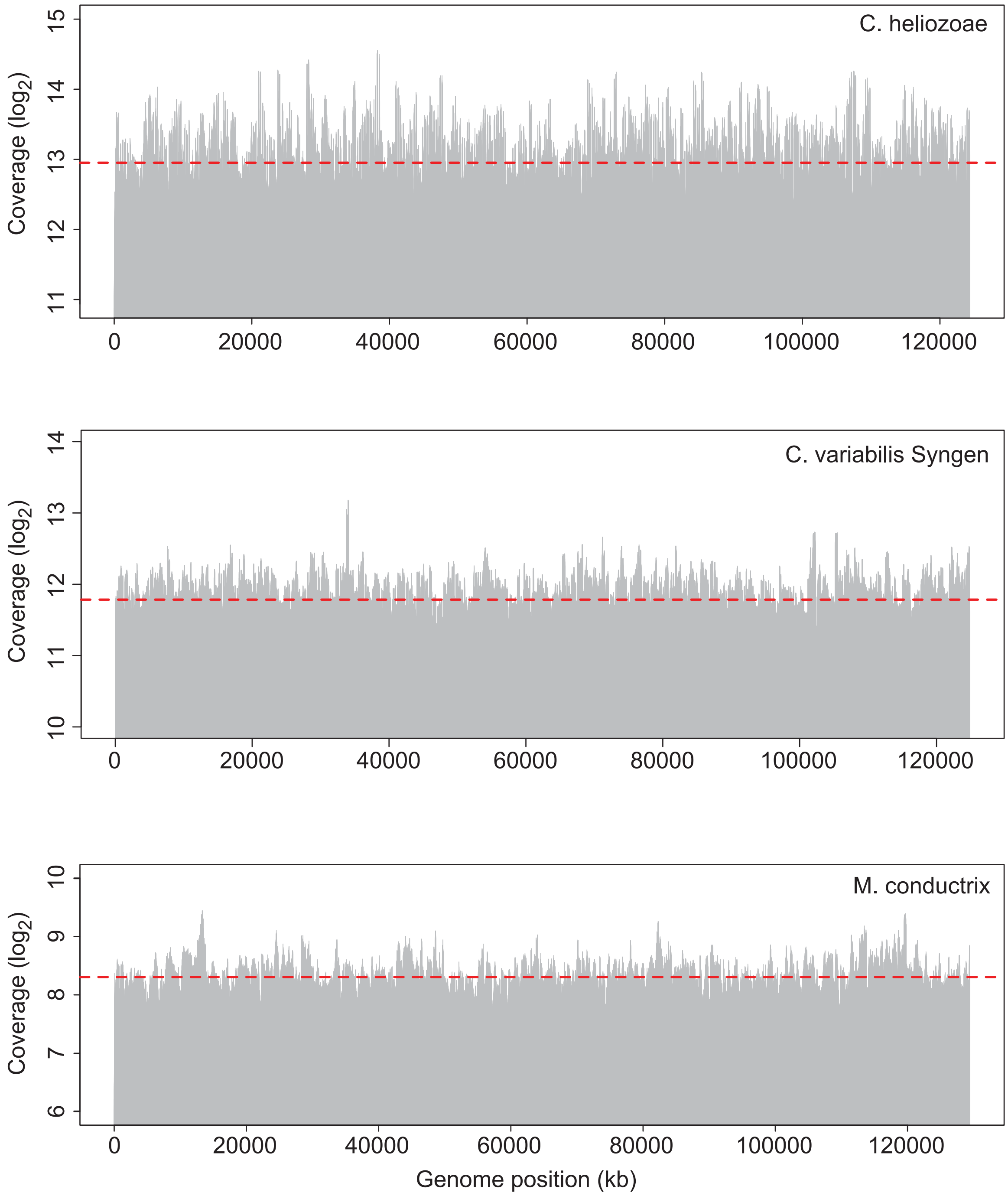

Supplement: Supplementary file 1 — Supplementary Information [file 41598_2017_10388_MOESM1_ESM.pdf]
